# Quorum sensing of *Streptococcus mutans* is activated by *Aggregatibacter actinomycetemcomitans* and by the periodontal microbiome

**DOI:** 10.1186/s12864-017-3618-5

**Published:** 2017-03-20

**Authors:** Szymon P. Szafrański, Zhi-Luo Deng, Jürgen Tomasch, Michael Jarek, Sabin Bhuju, Manfred Rohde, Helena Sztajer, Irene Wagner-Döbler

**Affiliations:** 1 Microbial Communication, Helmholtz-Center for Infection Research, Braunschweig, Germany; 2 Genome Analytics, Helmholtz Centre for Infection Research, Braunschweig, Germany; 3 Central Facility for Microscopy, Helmholtz Centre for Infection Research, Braunschweig, Germany; 4 Present address: Hannover Medical School (MHH), Hannover, Germany

## Abstract

**Background:**

The oral cavity is inhabited by complex microbial communities forming biofilms that can cause caries and periodontitis. Cell-cell communication might play an important role in modulating the physiologies of individual species, but evidence so far is limited.

**Results:**

Here we demonstrate that a pathogen of the oral cavity, *Aggregatibacter actinomycetemcomitans* (*A. act.*), triggers expression of the quorum sensing (QS) regulon of *Streptococcus mutans,* a well-studied model organism for cariogenic streptococci, in dual-species biofilms grown on artificial saliva. The gene for the synthesis of the QS signal XIP is essential for this interaction. Transcriptome sequencing of biofilms revealed that *S. mutans* up-regulated the complete QS regulon (transformasome and mutacins) in the presence of *A. act*. and down-regulated oxidative stress related genes. *A.act*. required the presence of *S. mutans* for growth. Fimbriae and toxins were its most highly expressed genes and up-regulation of anaerobic metabolism, chaperones and iron acquisition genes was observed in co-culture. Metatranscriptomes from periodontal pockets showed highly variable levels of *S. mutans* and low levels of *A. act.*. Transcripts of the alternative sigma-factor SigX, the key regulator of QS in *S. mutans*, were significantly enriched in periodontal pockets compared to single cultures (log_2_ 4.159, FDR ≤0.001, and expression of mutacin related genes and transformasome components could be detected.

**Conclusion:**

The data show that the complete QS regulon of *S. mutans* can be induced by an unrelated oral pathogen and *S. mutans* may be competent in oral biofilms in vivo.

**Electronic supplementary material:**

The online version of this article (doi:10.1186/s12864-017-3618-5) contains supplementary material, which is available to authorized users.

## Background

*Streptococcus mutans* (*S. mutans*) has been isolated from caries lesions in 1924 and was consequently considered the etiological agent of caries, consistent with Koch`s postulates [1]. Its physiological properties were since then analysed in great detail and were in accordance with a role as primary causative agent of cavities [2]. Cultivation independent studies, particularly metagenome and metatranscriptome analyses, have completely revolutionized our understanding of oral diseases like caries and periodontitis. They clearly show that *S. mutans* is only a tiny component of the active oral microbiome. Even in caries lesions, it accounts for less than 1% of the active microorganisms [3, 4] and the onset of early childhood caries can be predicted by the presence of *Prevotella* spp*.* but not *S. mutans* [5]. The etiology of caries is therefore one of the prime examples for the paradigm shift from Koch´s postulates towards the concept of a polymicrobial origin of disease caused by a shift in the composition and metabolic activity of a complex community towards dysbiosis [6]. It is thus important to understand the mechanisms that contribute to dysbiosis development and this will provide novel concepts for treatment [6]. For example, modifying the interaction pattern could potentially have complex downstream effects on the community [7].

Cell-cell communication might be one of the mechanisms that can tip the balance between health and dysbiosis [8]. Oral biofilms are hot spots of cell-cell communication due to the high species diversity, the physical contact between adjacent cells, and large metabolic activity of the community [9]. *S. mutans* is a good model organism for studying the role of QS in oral biofilms because the functioning of its QS system is relatively deeply understood and some of its characteristics are conserved. In streptococci, short hydrophobic peptides induce competence via a RGG type transriptional regulator [10]. These peptides have been shown to mediate interspecies communication [11], and have been exploited for antibacterial strategies [7, 12, 13]. Communication can be induced by environmental signals and change the resistance of the bacteria towards cell wall degrading lysozyme [14]. However, it is currently not known if streptococci, or *S. mutans* in particular, are competent in their natural habitats in vivo, i.e. in the human or animal host. Metagenomics is not informative in this respect, since it only shows the presence of the DNA, but competence is a strongly regulated phenotype. Sequencing of the total mRNA of the microbial community provides information which genes *S. mutans* is actively transcribing in vivo. Therefore we searched the published metatranscriptomes of periodontal pockets [15–18] for transcripts from *S. mutans* and *A. act.* and investigated if the competence regulator SigX and downstream genes are expressed in the natural biofilm under the prevailing conditions and in the presence of the complex oral microbial community.

*S. mutans* synthesizes two quorum sensing (QS) signals, competence inducing peptide (CSP) and sigX inducing peptide (XIP) that are sensed through a two-component signal system (ComDE) and a Rgg-type intracellular transcriptional regulator (ComR), respectively [19]. Upon activation of ComR through binding of XIP, transcription of the alternative sigma-factor SigX is induced [19]. We recently showed that CSP controls transcription of mutacins only, thus we suggest to rename it mutacin inducing peptide (MIP) [20]. The regulon of SigX in *S. mutans* includes not only the transformasome machinery mediating genetic competence, but also mutacins whose transcription is controlled by the ComE response regulator [20]. Thus SigX is the central regulator of QS in *S. mutans*. It is not known if *sigX* is expressed in vivo. Moreover, natural conditions under which MIP is synthesized are unknown. We previously demonstrated induction of *sigX* by the human pathogenic fungus *Candida albicans*; both transformasome and mutacins were upregulated in co-culture, in accordance with the central role of SigX in *S. mutans* [20, 21].

Here we now ask if inter-kingdom communication between *S. mutans* and *C. albicans* is an exception, or if *S. mutans* might be able to communicate with other members of the oral microbiome. We studied its interaction with *Aggregatibacter actinomycetemcomitans* (*A. act).* This pathogen has been regarded as etiological agent of aggressive periodontitis for decades, but today is found to be a minor component of the oral cavity [22] while periodontitis is regarded as a polymicrobial infection [23], similar to the situation with *S. mutans* and dental caries. For species to interact in the oral cavity, their co-localization in vivo is essential. Only recently has it been possible to observe such co-localization with genus-level taxonomic resolution. In dental plaque samples, interestingly, A*ggregatibacter* spp*.* and *Streptococcus* spp*.* were found as dense aggregates at the tips of abundant hedgehog structures [24]. Thus studying interactions between *S. mutans* and *A. act.* might reveal mechanisms contributing to biofilm development in the oral cavity.

*A. act.* is doomed to co-operate. It entirely depends on other oral microorganisms to grow in saliva [25]. A “food for detoxification” interaction has been unraveled between *A. act*. and the commensal *S. gordonii* [26]. *S. gordonii* excretes lactic acid, which *A. act.* uses as a carbon source, and hydrogen peroxide, which *A. act.* detoxifies, and in a murine abcess model both species are more virulent together than alone [27]. Cocultivation with *S. gordonii* triggers expression of the complement resistance protein ApiA in *A. act.* and in such a way increases its resistance to host innate immunity [28].

Here we analysed the interaction between *S. mutans* and *A. act*. in dual-species biofilms. We monitored induction of SigX of *S. mutans* using a P_sigX_-GFP reporter strain [29]. To determine through which route *A. act.* might communicate with *S. mutans* we deleted key genes of *S. mutans*. We analysed the transcriptomes of both species using RNA sequencing. Finally we asked if competence and mutacin synthesis might be expressed by *S. mutans* also in oral biofilms in vivo. To this end we extracted its transcripts from metatranscriptomes of periodontal pockets and analysed the expression of the SigX regulon.

## Methods

### Strains, culture conditions and media

Microorganisms used in this study are listed in Additional file 1. *A. act.* ATCC 33384 was used in all experiments except for RNA sequencing where the “rough” strain HK1651 (CCUG 56173, Göteborg, Sweden) was used because it has a fully assembled and annotated genome. All cultures were incubated aerobically at 37 °C with 5% CO_2_ without shaking. Pre-cultures of *S. mutans* and *A. act*. strains were grown in Todd-Hewitt broth supplemented with yeast extract (THBY) (Becton, Dickinson and Company, Sparks, MD, USA). For the *sigX* reporter strain and deletion mutants, erythromycin (3 μg ml^-1^ and 10 μg ml^-1^, respectively) was added. Biofilms were cultivated in YNBB2 medium which contains 6.7 g l^-1^ yeast nitrogen base (YNB) synthetic medium (Difco Laboratories, Detroit, Mi, USA), 75 mM Na_2_HPO_4_–NaH_2_PO_4_ (pH 7.3), casamino acids (2 g l^-1^, Becton, Dickinson and Company) and sucrose (5 g l^-1^). The pH during biofilm growth was in the range 7.0–6.4. Biofilms grown on modified chemically defined artificial saliva medium (artificial saliva) [30] were used for RNA sequencing. Mucin was omitted from the medium, haemin was replaced with 1.2 μM FeCl_3_, 14.6 mM sucrose was added and the medium was buffered with 75 mM Na_2_HPO4–NaH_2_PO_4_ (pH 7.3). In some experiments, chemically defined mineral medium CDM [31] was used. For the construction of the *hdrRM*, *brsRM* and *liaS* deficient strains, the PCR ligation mutagenesis approach [32] was used. Primers are listed in Additional file 1: Table S1. Mutants were verified by PCR and sequencing.

### Biofilm analysis

Crystal violet staining of biofilms, DAPI staining, fluorescence microscopic analysis and field emission scanning electron microscopy were performed as described [21]. For quantitative PCR, extracellular DNA was removed from the biofilms, genomic DNA was extracted and *S. mutans* genomes were quantified as described [21]. Quantification of *A. act*. genomes was performed using forward primer GGACGGGTGAGTAATGCTTG and reverse primer CC**T**TTACCCCACCAACTACC and the annealing temperature was 58 °C (modified from [33]). To convert nanograms of DNA to numbers of cells, 2.222 fg was used as the weight of the genome of *A. act.*.

### Preparation of conditioned media and reporter strain biofilm assay

Culture supernatants were withdrawn, centrifuged (5000 rpm, 20 min, 4 °C), sterile filtered (0.22 μm, Roth, Karlsruhe, Germany) and frozen at -20 °C. *S. mutans* reporter strain SMP_*sigX*_GFP was grown in black Nunc 96 Well Optical Bottom Plates (Nunc, Langenselbold, Germany) in YNBB2 medium for 8 h. Cultivation media were removed and conditioned media (warmed to 37 °C) were loaded onto the reporter strain biofilms. Biofilms were further incubated for 3 h, supernatants were removed and the induction of *sigX* was measured as described [21].

### RNA isolation and sequencing

Biofilms of *S. mutans*, *A. act.* or both were cultivated in 24 well cell culture plates (Greiner Bio-One, Frickenhausen, Germany) for 4 to 24 h. After removal of the supernatants, biofilms were covered with 300 μl RNA Protect (Qiagen, Hilden, Germany). The biofilms were scraped off with sterile one-way cell scrapers, transferred to sterile Eppendorf tubes, centrifuged (4 °C, 5 min, 13,000 rpm), the supernatant was removed and the pellets were stored at -70 °C until RNA extraction. For RNA extraction the pellets were suspended in 0.2 ml lysis buffer containing 10 mM Tris, 1 mM EDTA (pH 8.0), 2.5 mg/ml lysozyme (Sigma-Aldrich, Taufkirchen, Germany) and 50 U/ml mutanolysin (Sigma-Aldrich, Taufkirchen, Germany) and incubated at 25 °C for 1.5 h at 350 rpm. Subsequently 1 ml TRIzol reagent (Invitrogen, Carlsbad, CA, USA) was added, samples were mixed and incubated at room temperature (RT) for 5 min. Cells were then vortexed for 30 s in the presence of 50 μg sterile, acid-washed glass beads (diameter 106 μm; Sigma-Aldrich, Taufkirchen, Germany). This was repeated 10 times with 1 min intervals on ice. Samples were briefly centrifuged, supernatants were transferred to new tubes and vortexed with ice cold 1-bromo-3-chloropropane (Sigma-Aldrich, Taufkirchen, Germany) for 15 s followed by 5 min incubation at RT. Samples were centrifuged (4 °C, 15 min, 13,000 rpm), the aqueous phase was collected, nucleic acids were precipitated with 1 volume of ice cold isopropanol, the solvent was removed and the pellet was suspended in double distilled autoclaved water. After removal of DNA with RNase free DNaseI (Qiagen, Hilden, Germany) using the in-solution digestion protocol, RNA was purified using the RNeasy mini kit (Qiagen, Hilden, Germany) according to the manufacturer`s instructions with additional DNaseI on-column digestion. Bacterial rRNAs were depleted using MicrobExpress (Ambion, Austin, TX). mRNA was converted to cDNA using ScriptSeq v2 RNA-Seq (Epicentre Biotechnologies, Madison, Wisconsin, USA). For sequencing 12 pM of each library was used and 8 samples were multiplexed on a single lane. Cluster generation was performed with cBot (Illumina) using a TruSeq SR Cluster Kit v3–cBot-HS (Illumina). Two biological replicas were sequenced for each treatment. In total two and half lanes (containing 20 libraries) were single-end sequenced for 50 cycles on an Illumina HiSeq 2500 sequencer using the TruSeq SBS Kit v3 - HS (Illumina). Image analysis and base calling were performed using the Illumina pipeline v 1.8. Sequencing data are available at Gene Expression Omnibus under accession number GSE75019.

### RNAseq data analysis

The *S. mutans* UA159 and *A. act.* HK1651 reference genomes (GenBank accession numbers NC_004350.2 and CP007502.1, respectively) were used. After clipping adapters and barcodes, reads were aligned with the reference genome using Bowtie 2 [34]. HTSeq-count was used to count the mapped reads per gene (http://www-huber.embl.de/users/anders/HTSeq). Differential gene expression was calculated using the R package edgeR [35]. Genes showing differential expression in dual-species biofilms (all time points compared to the 4 h time point, log_2_ fold change < - 2 and > 2; *p* < 0.01, were sorted into clusters using the c-means algorithm [36]. The Rockhopper software was used to identify novel transcripts and determine operons [37]. PRIMER & PERMANOVA+, (PRIMER-E, Plymouth, UK) were used to perform PCA analysis based on standardized log-transformed sequence abundances grouped to genes. The R package pheatmap was used to create heatmaps [38].

## Results

### Induction of the alternative sigma-factor SigX of *S. mutans* by *A. act*

**Fig. 1:**
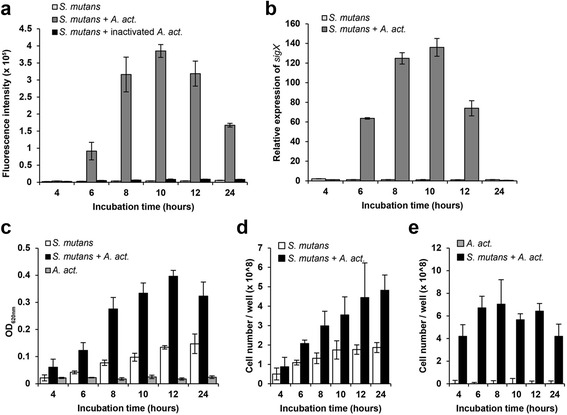
The alternative sigma-factor *sigX* of *S. mutans* is induced by *A. act.* when both species are co-cultured in a biofilm. **a** *sigX* expression determined by fluorescence intensity of *S. mutans* SMP_*sigX*_GFP, a gfp-reporter for *sigX* expression*.* **b** *sigX* expression determined by q-RT-PCR. The data were normalized to *sigX* expression in a *S. mutans* biofilm after 4 h. **c** Growth of *S. mutans* and *A. act.* in single- and dual-species biofilms determined by crystal *violet* staining. **d** Cell numbers of *S. mutans* and (**e**) *A. act* in single- and dual-species biofilms determined by q-PCR of the 16S rRNA gene. Mean and standard deviation of three independent experiments are shown for **a**–**c**, and of four independent experiments with two technical replicates for **d**–**e**

**Fig. 2:**
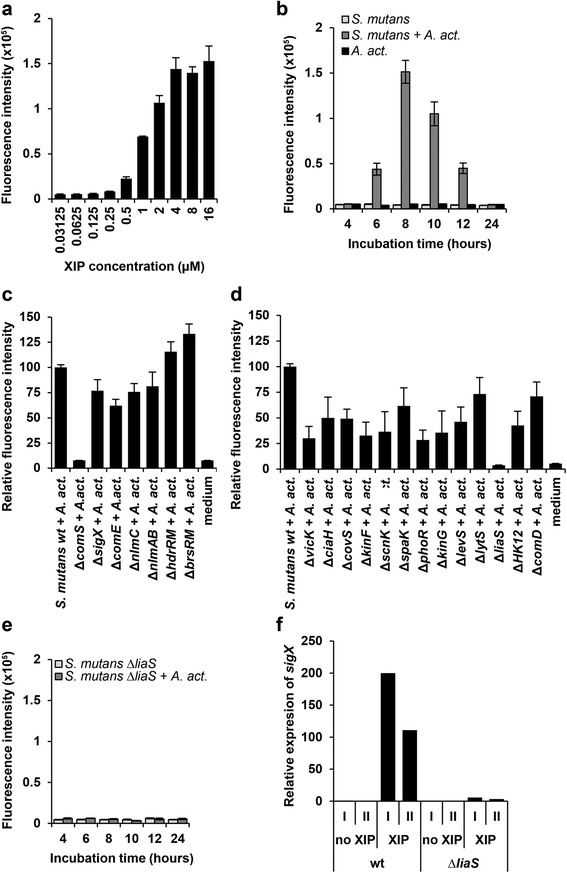
Activation of the *sigX* promoter by culture supernatants. Test biofilms (8 h old) of the reporter strain *S. mutans* SMP_*sigX*_GFP were used to test the activity of culture supernatants. Fluorescence intensity was determined after 3 h of incubation. **a** The reporter biofilm detected XIP in the medium above a concentration of 0.5 μM. **b** Fluorescence in the reporter biofilm was induced by culture supernatants from biofilms of *S. mutans* and *A. act.* cultivated together for 6 – 12 h, but not from single species biofilms or dual species biofilms at 4 h and 24 h of co-cultivation. **c** The reporter biofilm was challenged with culture supernatants from dual special biofilms of *A. act* with mutants of *S. mutans* carrying deletions of QS genes. Deletion of *comS* rendered culture supernatants inactive. **d** Effect of deletions of histidine kinases from two-component signal transduction systems of *S. mutans* on the activity of culture supernatants from biofilms with *A. act*. Deletion of *liaS* renders culture supernatants inactive. **e**-**f** Analysis of the Δ*liaS* mutant of *S. mutans*. **e** Culture supernatants from biofilms of *S. mutans* Δ*liaS* and *A. act.* cultivated separately or together for 4 – 24 h did not induce fluorescence in the reporter biofilm. **f** XIP induced expression of *sigX* in the wild-type *S. mutans*, but not in the Δ*liaS* mutant. Expression of sigX was determined by q-RT-PCR of *sigX* in the absence or presence of 0.5 μM XIP. The data were normalized to *sigX* expression in an uninduced *S. mutans* wild-type biofilm. Mean and standard deviation of three experiments are shown for **a**–**e**, and of two independent experiments (I and II) in **f**

To determine if *A.act.* is able to induce QS in *S. mutans* we co-cultured it with a reporter strain. *S. mutans* SMP_*sigX*_GFP carries a plasmid where the promoter of the alternative sigma-factor *sigX*, the master regulator of both competence and mutacin synthesis, is fused to GFP [29]. Green fluorescence was measured during 24 h of biofilm growth. In the absence of a co-cultivation partner, no induction of fluorescence was observed in *S. mutans* (Fig. 1a). By adding the autoinducer XIP (*sigX* inducing peptide), strong fluorescence could be induced (Fig. 2a), indicating that extracellular XIP could be imported into the cells in this medium [39]. Live, but not heat inactivated *A. act.* strongly induced *sigX* expression in dual-species biofilms (Fig. 1a). Fluorescence showed a peak after 10 h of biofilm growth and declined subsequently. Quantification of *sigX* expression by q-RT-PCR (Fig. 1b) similarly showed a peak at 10 h in co-culture.

Both species grew better together than alone. Biofilm biomass increased continuously for *S. mutans* biofilms, but much stronger in the dual-species biofilm of *S. mutans* with *A. act* (Fig. 1c). At every time point the biomass of dual-species biofilms was at least twice that of single-species biofilms. *A.act.* did not form biofilms when grown alone, only small cell aggregates attached to the bottom of the wells were observed. q-PCR of the 16S rRNA gene revealed that cell numbers of *S. mutans* increased throughout the experiment and reached ~2 × 10^8^ cells/well in mono-culture and more than twice that amount (~4.5 × 10^8^ cells/well) in dual-species biofilms (Fig. 1d). *A.act.* reached ~ 6 – 8 × 10^8^ cells/well in dual-species biofilms, but did not grow in single culture (Fig. 1e). The numbers of *A.act.* did not change significantly in dual species biofilms during the 6–12 h period. This implies that the increase in biofilm biomass during this time frame (Fig. 1c) corresponded to the increase in numbers and extracellular polysaccharides (EPS) of *S. mutans*.

### The alternative sigma-factor SigX is activated by an extracellular compound in dual-species biofilms

To test whether a compound excreted into the medium might be responsible for the induction of *sigX* we applied sterile-filtered supernatants from single- and dual-species biofilms (cultivated for 4 and 24 h) to biofilms of the *S. mutans* SMP_*sigX*_GFP reporter strain grown for 8 h; fluorescence intensity was determined after additional 3 h of incubation. Under these conditions, the reporter strain detected XIP concentrations above 0.25 μM (Fig. 2a). Supernatants of dual-species biofilms that had been cultivated between 6 and 12 h activated P_*sigX*_, whereas mono-culture supernatants had no effect, irrespective of how long they had been cultivated (Fig. 2b). The data show that it is a component in the culture supernatant that activates the *sigX* promoter, and that this component is not present in single species biofilm supernatants of either *S. mutans* or *A. act*. Therefore, it must be the result of an interaction between those two organisms. The time-course of activation of the reporter strain by dual-species culture supernatants is in complete agreement with the time course of fluorescence and *sigX* expression observed in dual-species biofilms (Fig. 1a, b).

### Activation of SigX requires the autoinducer synthase ComS from *S. mutans*

To investigate which regulatory systems of *S. mutans* might be involved in mediating the induction of *sigX*, we tested culture supernatants from deletion mutants of *S. mutans* (Additional file 1) co-cultivated with *A. act*.. Fig. 2c shows that in the absence of the autoinducer synthase ComS, an active compound could not be produced in dual-species biofilms. ComS synthesizes a pre-peptide that is cleaved and exported to yield the mature XIP autoinducer. By contrast, neither SigX nor the response regulator ComE and the mutacins NlmC and NlmAB are required to produce an active supernatant in co-culture with *A. act..* Interestingly, the activity of the supernatants was slightly higher in biofilms of *A. act.* with *S. mutans* lacking the two gene regulatory systems *HdrRM* or *BrsRM*.

The data suggest that the autoinducer synthase ComS of *S. mutans* is stimulated by an extracellular component produced in co-culture with *A. act.* to synthesize the XIP pre-peptide, which after export and cleavage needs to be imported again and can then induce transcription of *sigX* and downstream genes. Since this re-import of XIP in *S. mutans* is abolished in rich medium [39], we tested the influence of different media on the induction of *sigX* in dual-species biofilms. *SigX* induction was only observed in peptide-free CDMS medium and in YNBB2, but not in THBYS or artificial saliva (AS) (Additional file 2), although the biofilms grew on all media (Additional file 3). Interestingly, mucin inhibited *sigX* activation in a concentration dependent manner (Additional file 4a). It was therefore omitted from the artificial saliva medium used for transcriptome analysis.

### Activation of SigX requires the histidine kinase *liaS* from *S. mutans*

We then tested the influence of the histidine kinases (HKs) from the 13 two-component signal transduction systems of *S. mutans* on the activity of dual-species biofilm supernatants. Supernatants from *A. act.* grown in dual-species biofilms with deletion mutants of *S. mutans* lacking HKs were less active than those from *S. mutans* wild-type, most likely because the mutants have growth defects [40], but with one exception all of them were active (Fig. 2d). However, the supernatant from the Δ*liaS* mutant dual species biofilm was inactive over the full 24 h of growth with *A. act.* (Fig. 2e). This was not caused by impaired growth (data not shown). Subsequently, we tested the response of *S. mutans ΔliaS* to exogenous XIP. Mono-species biofilms of wild-type and *S. mutans ΔliaS* were cultivated with and without XIP for 8 h and the expression of the *sigX* gene was investigated using q-RT-PCR. In the wild-type, the expression of *sigX* was 200fold or 100fold induced by XIP, while it was only 2-5fold induced in the *ΔliaS* mutant (Fig. 2f), suggesting that this mutant is unable to import the XIP signal from the extracellular medium into the cell.

### Transcriptome analysis of *S. mutans* and *A. act* in dual-species biofilms

We applied RNA sequencing to investigate the transcriptional activities of both *S. mutans* and *A. act* in dual species biofilms. Modified artificial saliva was used as cultivation medium to mimick the conditions in the oral cavity. We used the highly leukotoxic and adhesive “rough” strain *A. act*. HK1651 [41] because its fully annotated genome is available. It differs in terms of serotype, morphotype and amount of toxins produced from the type strain. HK1651 grew together with *S. mutans* in a similar way as the type strain of *A. act*. and it also activated *sigX*, although with a slightly shorter duration in comparison to the type strain (Additional file 4b,c).

**Fig. 3:**
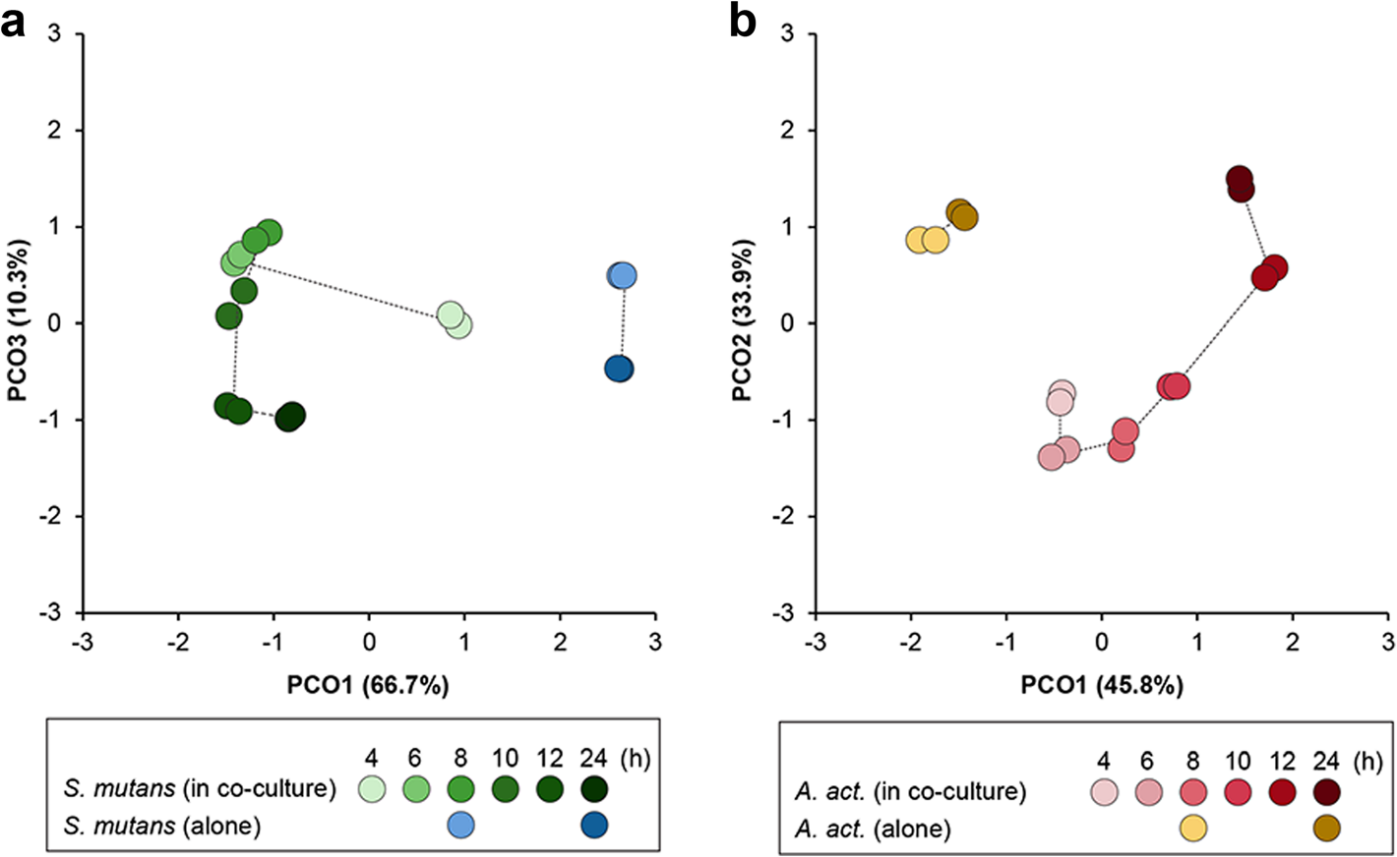
Principal Component Analysis of single- and dual-species biofilm transcriptomes. **a** Transcriptomes of *S. mutans* grown in single-species biofilms (*blue*) and in dual-species biofilms with *A. act*. (*green*). **b** Transcriptomes of *A. act*. in single-species biofilms (*red*) and in dual-species biofilms with *S. mutans* (*yellow*). Standardized log_2_-transformed read counts were used in these analyses

We analyzed the transcriptome of both species in dual-species biofilms during (8 h) and after (24 h) activation of *sigX* in comparison to gene expression in mono-species biofilms. In addition the transcriptomes of both species in dual-species biofilms were analyzed at 6, 8, 10, 12 and 24 h of growth and compared to the expression at 4 h. In total, 513.4 million reads were obtained, providing on average 11 and 1.1 million reads per sample. 93% (*S. mutans*) and 84% (*A. act.*) of the expressed genes had a mean count per gene per million reads (CPM) higher than 10 (Additional file 4d-f). Using Rockhopper we identified 434 operons and predicted 204 small RNAs (including 71 antisense RNAs) in the *S. mutans* genome (Additional file 5). Principal Component Analysis (PCA) (Fig. 3) showed a good correlation between replicates and clearly separated single- and dual-species biofilm transcriptomes. Moreover, gradual changes in co-culture transcriptomes of each species could be observed during the 24 h of growth and expression profiles could be separated into several clusters clusters and groups of clusters (Additional files 6 and 7).

### Comparison of transcriptomes of *S. mutans* grown alone and with *A. act*

**Fig. 4:**
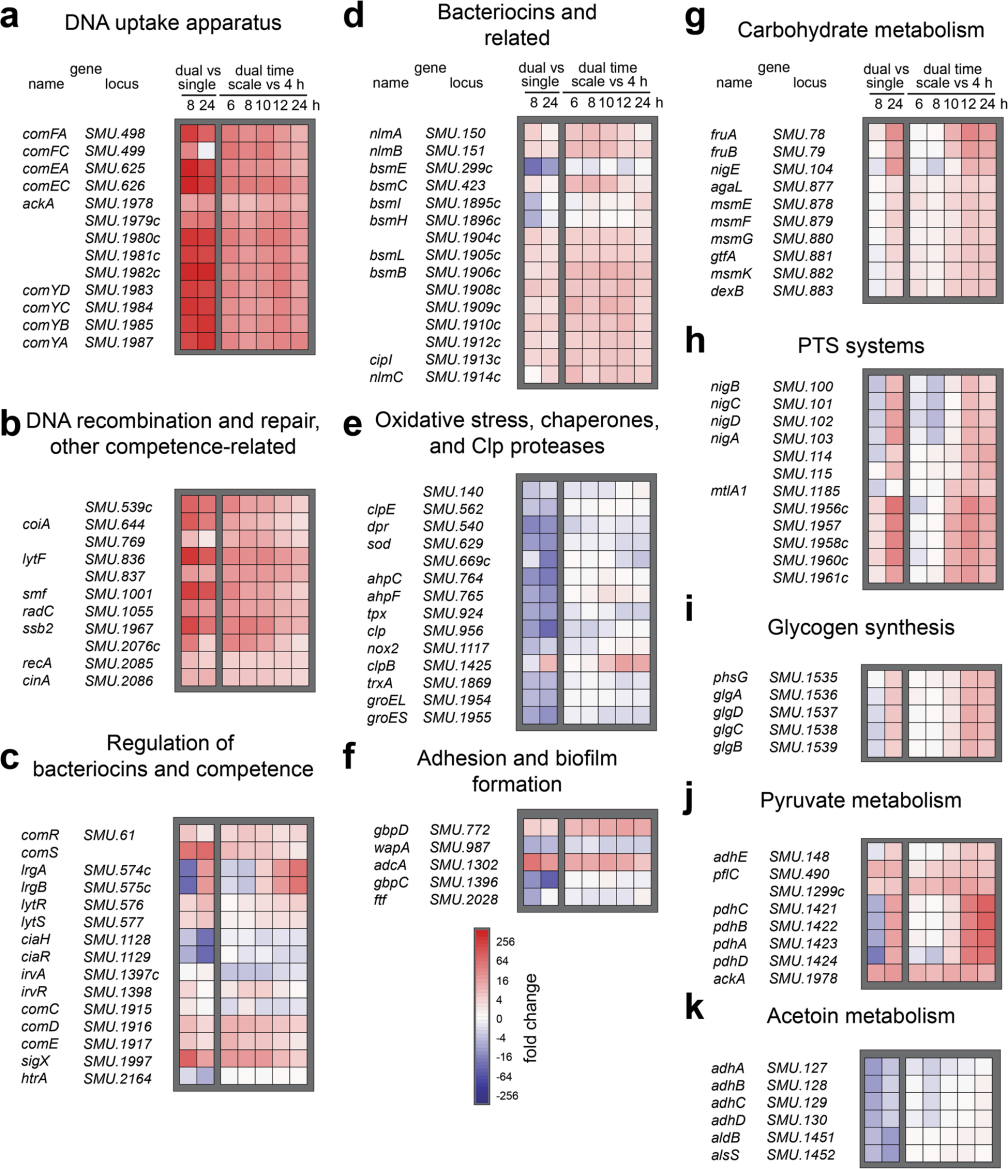
Transcriptional activity of *S. mutans* alone and in co-culture with *A. act*.. Heatmaps show relative gene expression of *S. mutans* after 8 and 24 h of biofilm growth in dual species biofilms with *A. act*. compared with the expression in single-species biofilms (*left two columns*) or after 6, 8, 10, 12 and 24 h of biofilm growth in dual species biofilms compared with gene expression at 4 h (*right five columns*). Selected genes were significantly differentially expressed in at least one condition (FDR ≤ 0.05) and are grouped according to function in panel **a**-**k** andordered based on their position in the *S. mutans* chromosome

Comparative analysis of biofilm transcriptomes of *S. mutans* grown alone and with *A. act*. yielded 260 and 246 differentially expressed genes (log_2_ fold change ≤ - 2 and ≥ 2; *p* < 0.01) for the 8 h and 24 h time points, respectively. The complete SigX regulon was induced in dual-species biofilms, including the DNA uptake apparatus (Fig. 4a) and the recombination machinery (Fig. 4b) suggesting that in co-culture *S. mutans* became genetically competent. The alternative sigma-factor *sigX*, the central regulator of QS, was among the most abundant transcripts and revealed a 72 fold change at 8 h (Fig. 4c). Accordingly, its primary target, the gene encoding the pre-peptide synthase ComS, was also highly upregulated. The ComDE two-component system that directly controls mutacin synthesis was slightly up-regulated, while the QS modulating CiaRH and the membrane bound protease HtrA were down-regulated (Fig. 4c). Mutacins also belong to the SigX regulon [20] and accordingly they were slightly up-regulated (Fig. 4d). Oxidative stress related genes, e.g. superoxide dismutase and chaperones, were repressed (Fig. 4e). Of the genes related to biofilm formation and adhesion, e.g. glucan binding proteins, some were up- and some were down-regulated (Fig. 4f). Carbohydrate metabolism of *S. mutans* most likely differed in the co-culture. Genes coding for exo-fructosidases, alpha-glucosidase and multi-sugar utilization enzymes (Fig. 4g) as well as sugar transport systems (Fig. 4h) were induced. Glycogen synthesis was induced especially at the later time-points (Fig. 4i). The expression of pyruvate dehydrogenase decreased in 8 h co-culture and was strongly induced at 24 h (Fig. 4j).

### Comparison of transcriptomes of *A.act.* grown alone and with *S. mutans*

**Fig. 5:**
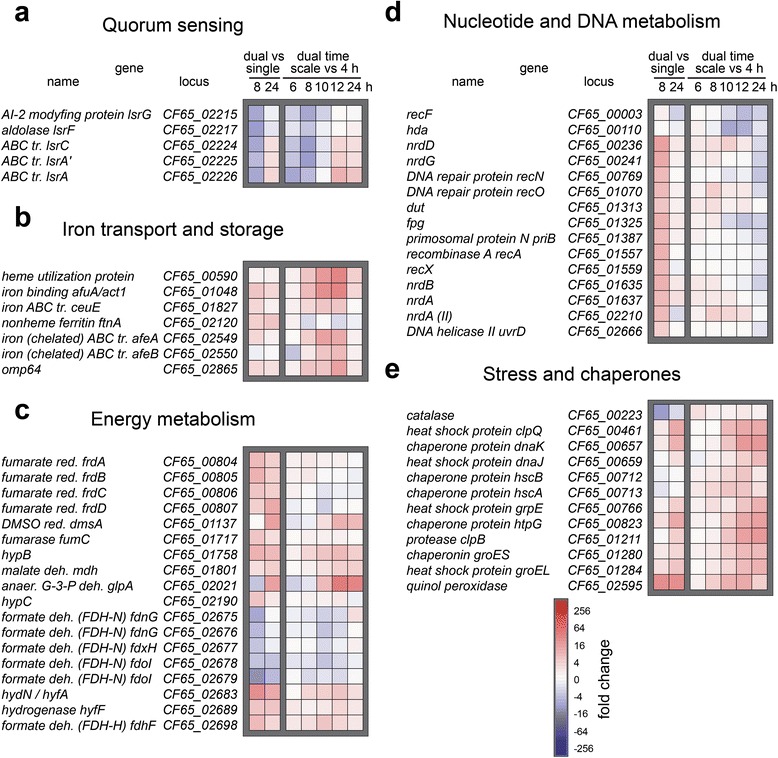
Transcriptional activity of *A. act.* alone and in co-culture with *S. mutans*. Heatmaps show relative gene expression of *A. act.* after 8 and 24 h of biofilm growth in dual species biofilms with *S. mutans* compared with the expression in single-species biofilms (*left two columns*) or after 6, 8, 10, 12 and 24 h of biofilm growth in dual species biofilms compared with gene expression at 4 h (*right five columns*). Selected genes are grouped according to function in panel **a**-**e** and ordered based on their position in the *A. act.* chromosome

Comparative analyses of transcriptomes of *A. act.* grown with *S. mutans* yielded 170 and 97 differentially expressed genes (log_2_ fold change ≤ -2 and ≥ 2; *p* < 0.01) for 8 h and 24 h, respectively. The main virulence factors of *A.act.* showed constant high expression irrespective of the tested condition. Leukotoxin was the second most highly expressed *A. act*. transcript (Additional file 5). The tight adherence (*tad*) locus that is required for the assembly of adhesive pili made up 21% of the total *A. act.* transcriptome and *flp-1* was the most abundant transcript overall. The *lsrACFG* genes coding for an AI-2 transporter and AI-2 metabolizing enzymes were 8 fold down-regulated in the 8 h old co-culture (Fig. 5a). The autoinducer synthase LuxS was not differentially expressed. Iron uptake genes were up-regulated in co-culture (Fig. 5b) and changes in anaerobic metabolism likely occured: Fumarate reductase, malate dehydrogenase, formate dehydrogenase (FDH-H) and formate hydrogenlyase complex, and subunits of hydrogenase were induced, while the expression of formate dehydrogenase from the nitrate reductase complex (FDH-N) decreased (Fig. 5c). Genes coding for enzymes involved in nucleotide and DNA metabolism were induced especially in 8 h old dual species-biofilms (Fig. 5d) and expression of chaperones and heat shock proteins was up-regulated in the 24 h old co-culture. Quinol peroxidase [42] was most strongly (22-fold) induced in both 8 and 24 h co-culture biofilms (Fig. 5e). This enzyme reduces H_2_O_2_ to water using reducing equivalents from quinol, a component of the respiratory chain. The complement resistance protein ApiA (annotated as putative adhesion/invasion in strain HK1651) and catalase were 6-fold and 11-fold down-regulated at 6 h in co-culture (Fig. 5e).

### Changes in the transcriptomes of *S. mutans* and *A. act*. during growth in dual-species biofilms for 24 h

We found 316 and 309 genes of *S. mutans* and *A. act*., respectively, to be differentially expressed during growth in co-culture compared to their expression at 4 h. In *S. mutans* the competence and mutacin related genes were induced from 6 h onwards (Fig. 4a-d). From 10 h onwards the reprogramming of metabolism occurred (Fig. 4g-j). In *A. act.* the AI-2 metabolizing enzymes were repressed between 6 and 8 h (Fig. 5a) and induced at later time-points, in accordance with the up-take of AI-2 in late stationary phase during nutrient depletion. Iron sequestering was most strongly induced between 6 and 8 h (Fig. 5b). We observed the induction of stress response proteins, chaperones, and the ClpB protease (Fig. 5e).

### Expression of the quorum sensing regulon of *S. mutans* in periodontal pockets

Additional files 8 and 9 show the total and relative number of transcripts from *S. mutans* and *A. act*, respectively, in four metatranscriptome studies of the periodontal pocket microbiome [16–18, 43]. Transcripts from both organisms were detected in all studies, although at low abundance in most samples. However, in some samples, 20 to 30% of all reads mapped to *S. mutans* UA159, showing an extremely strong contribution of this species alone to the community metatranscriptome in some patients. No clear correlation with health or disease was found, yet interestingly, *S. mutans* transcripts had the highest relative abundance in the study of Yost et al. who investigated periodontitis progression [18]. Transcripts from *A. act.* were rare in all periodontal metatranscriptomes; the maximum value was found in a sample from a healthy individual, where they accounted for 1.4% of all reads.

**Fig. 6:**
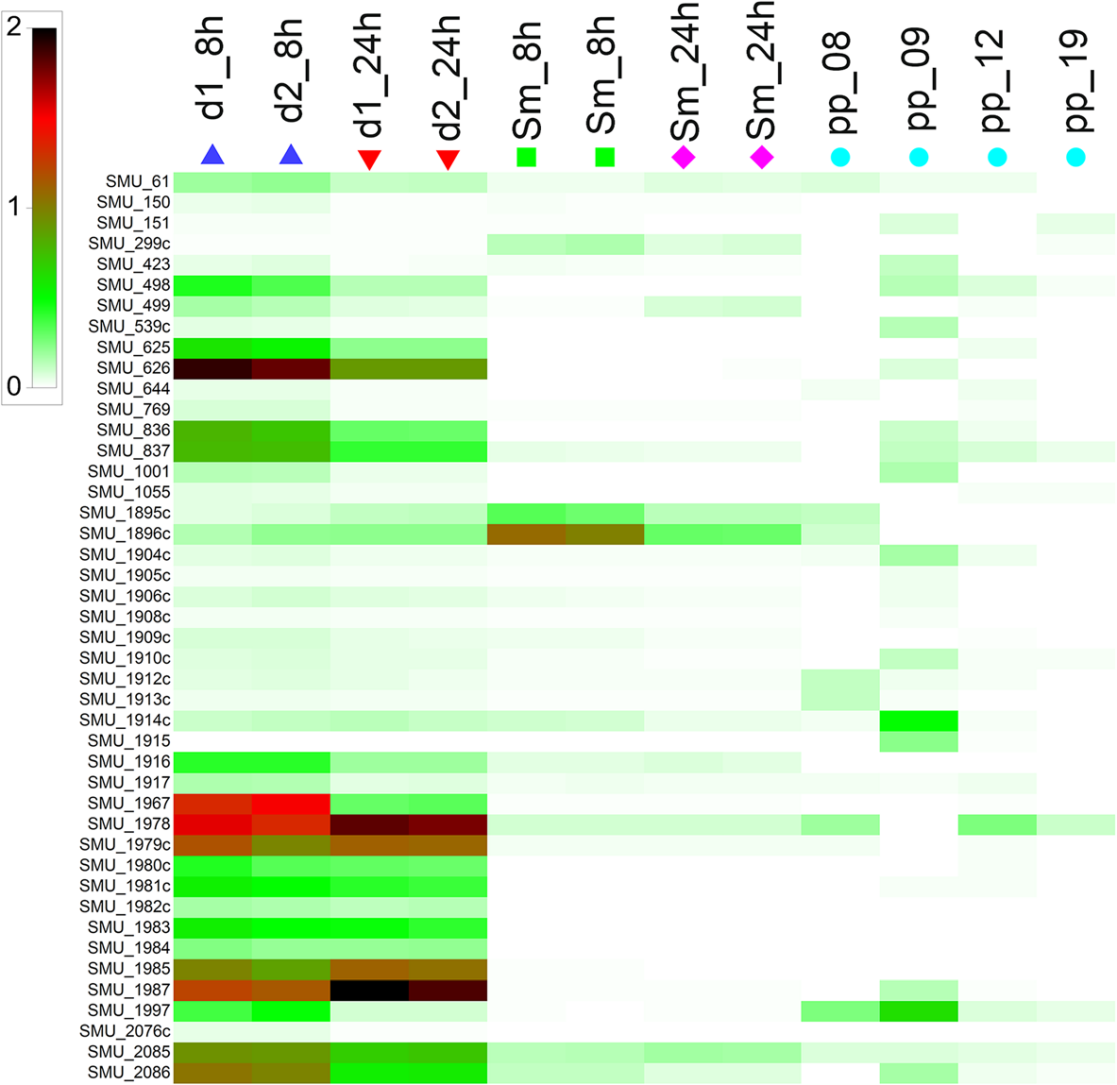
Expression of the QS regulon of *S. mutans* in dual culture, single culture, and in periodontal pockets. Relative abundance of transcripts of *S. mutans* (% of total transcripts) after 8 (d1_8h, d2_8h) and 24 h (d1_24h, d2_24h) in co-culture with *A. act.*, in single culture after 8 h (Sm_8h) and 24 h (Sm_24h), and in periodontal pockets (pp_08, pp_9, pp_12, pp_19). Sample pp_08 was derived from an individual with chronic periodontitis, while the other three periodontal pocket samples were derived from healthy individuals. RNAseq data from laboratory cultures are shown for two biological replicates. Metatranscriptome data from [16]

A detailed analysis was performed for samples with the highest numbers of reads (between 8347 and 37,798) which were derived from three healthy patients and one with chronic periodontitis [44]. The expression level of the 50 most highly expressed genes of *S. mutans* is shown in Additional file 5. The transcriptional profile depicts an actively growing population: Genes for important metabolic enzymes (pyruvate-formate lyase, glyceraldehyde-3-phosphate dehydrogenase, glycerol dehydrogenase, sugar phosphotransferase system, glucosyltransferase, alcohol-acetaldehyde dehydrogenase, 6-phosphofructokinase) are expressed, as well as genes encoding RNA polymerase, glucan branching enzyme, surface antigen SpaP and the ATP-dependent protease ClpE. The competence related genes with an average abundance of transcripts ≥0.01% is shown in Table 1. The most strongly expressed competence related gene was the master regulator of QS in *S. mutans*, the alternative sigma-factor *sigX* (SMU_1997), which had an average abundance of 0.23%. Additionally, transformasome as well as mutacin related transcripts could be detected. The most strongly expressed bacteriocin was NlmC (mutacin V), which is also the most highly expressed bacteriocin in pure culture. Interestingly, transcripts of *comC* (SMU_1915), encoding the synthase for the precursor of the mutacin inducing peptide MIP were also detected (0.06%). Not all components of the SigX regulon were found, but since the overall transcript level of *S. mutans* was relatively low, this is probably due to lack of sequencing depth. Because of the low absolute number of competence related transcripts in periodontal pockets, it was not possible to calculate the difference in gene expression for transcripts with average abundance <0.01%. Significant (FDR < 0.01) differential expression was found for *sigX*, *comFA*, *comC* and a hypothetical protein (Additional file 5). The result for *comC* should be interpreted with great caution, because in two of the periodontal pocket samples (AU_8 and AI_19) no transcript of *comC* was found, while in the other two (AU_9 and AU_12) their frequency was low (0.212% and 0.010% of all transcripts). However, it is now known that competence is induced by SigX, while CSP, the product of processed ComC, controls mutacin synthesis only and was therefore renamed MIP (mutacin inducing peptide) [20]. From all competence related genes, *sigX* was most strongly differentially enriched (log2 fold change 4.159, FDR < 0.001). A comparison of the expression of the QS related genes of *S. mutans* in dual and single species biofilms and in periodontal pockets based on percent of the respective transcript in comparison to the total amount of transccripts from this sample (Fig. 6) shows that biological replicates of in vitro cultures were almost identical, while periodontal communities showed large differences in the expression of QS related genes. However, *sígX* was not expressed in pure culture of *S. mutans*, neither during exponential growth nor in stationary phase, but was expressed in all four periodontal pockets, sometimes at levels similar (sample pp_8) or even higher (sample pp_09) than those in dual-species biofilms. Since SigX is the master regulator of both competence and bacteriocin synthesis, we therefore hypothesize that *S. mutans* is genetically competent in periodontal pockets.

**Table 1:** The expression of the SigX regulon of *S. mutans* in the periodontal community (sample AU_08, AU_09, AU_12, AU_19 from (39)

Locus tag	Mean percentage	Product
SMU_1997	0.23524	SigX, competence inducing alternative sigma-factor
SMU_1914c	0.12851	NlmC, bacteriocin
SMU_1978	0.12642	ComYA, transformasome component
SMU_837	0.05622	putative reductase, competence related
SMU_1915	0.05583	ComC, competence stimulating peptide precursor
SMU_2085	0.05410	RecA, recombination protein
SMU_1904c	0.05354	bacteriocin related
SMU_498	0.05178	ComFA, late competence protein
SMU_2086	0.05098	CinA, competence and damage inducible protein
SMU_1912c	0.03916	bacterocin related
SMU_1001	0.03679	Smf, DNA processing protein, competence related
SMU_1910c	0.03512	hypothetical protein
SMU_1987	0.03450	ATP-binding protein ComYA; late competence gene
SMU_539c	0.03360	signal peptidase type IV
SMU_61	0.03270	putative transcriptional regulator
SMU_836	0.03171	hypothetical protein
SMU_1913c	0.03148	immunity protein, BLpL-like
SMU_423	0.02861	hypothetical protein
SMU_151	0.02800	hypothetical protein
SMU_1895c	0.02739	hypothetical protein
SMU_1896c	0.02192	hypothetical protein
SMU_1917	0.01765	response regulator of the competence regulon, ComE;
SMU_626	0.01635	competence protein
SMU_644	0.01267	competence protein/transcription factor

Only genes with an average abundance > 0.01% are shown

## Discussion

We found that cell-cell communication in *S. mutans* was induced by the phylogenetically distantly related species *A. act*.. Induction occurred by a component in the culture medium that was only produced in live dual-species biofilms and that was not produced any more upon deletion of *comS* of *S. mutans*. Thus, production of the ComS prepeptide was induced in co-culture, similarly as in dual-species biofilms between *S. mutans* and the fungus *C. albicans* [21]. Leukotoxin and fimbriae are not likely to be involved since both the highly virulent strain HK1651 of *A. act*. and the “smooth” type strain induced QS in *S. mutans*. On the other hand, complex media containing components known to block the Opp permease required for import of the extracellular XIP [39] completely inhibited activation of SigX. This observation shows that the complete autocatalytic cycle of ComS synthesis, processing, export and import was activated in dual species biofilms. Since the stimulating factor is only produced in co-culture, it must be the result of an interaction between the two species in the biofilm. It is unclear if physical contact is required for the primary activation, yet since sterile filtered culture supernatants can activate SigX, this seems unlikely.

Deletion of the histidine kinase LiaS of *S. mutans* (Smu.486) resulted in a strain that could not be induced by *A. act.* or by synthetic XIP to express *sigX*. LiaS is a sensor histidine kinase which represses glucan binding protein C (*gbpC*) transcription and induces synthesis of mutacin IV (*cipB*) [45]. In our transcriptome data, *gbpC* was down-regulated, and mutacin IV was upregulated in dual-species biofilms with *A. act*.. Recently, the LiaR regulon was shown to be comprised of three genes, namely Smu.753, Smu.1727 and Smu.2084c [46]. None of them was differentially expressed in dual species biofilms, which makes it very unlikely that sensing of a specific stimulus was responsible for the effect of LiaS on *sigX* induction. Since our data show that import of externally added, synthetic XIP was not possible in a Δ*liaS* mutant, we hypothesize that the cell membrane of this mutant was not permeable for XIP any more. Therefore we further hypothesize that exported XIP cannot re-enter the cell and the positive feed-back loop for XIP synthesis cannot be triggered.

We show that mucin inhibited QS induction in dual-species biofilms in a concentration dependent way. Mucins are glycoproteins contributing to the antimicrobial activity of saliva [47], to which we can now add their ability to inhibit the QS of *S. mutans*. This might be caused by blockage of the Opp permease [39] that can then not import XIP anymore. Mucin concentration can be decreased in oral biofilms by streptococci [48] to levels which no longer block signaling.

*S. mutans* down-regulated the expression of genes encoding chaperones and genes involved in oxidative stress in co-culture with *A. act*. suggesting that *A. act*. protected *S. mutans* from oxidative stress. Oxygen may have been depleted by the aerobic respiration of *A. act*.. Surprisingly, its catalase gene was down-regulated in co-culture, and stress and chaperone related genes were up-regulated, in contrast to *S. mutans*. The strong up-regulation of the quinol peroxidase gene also indicates oxidative stress for *A.act.*. These apparent inconsistencies might be caused by oxygen gradients in biofilms [49] and by differences in the localization of the cells within the biofilm [24]. In dual species biofilms we observed tight balls of *A. act*. embedded in a thick layer of *S. mutans* cells (Additional file 10). Inside the balls, conditions were most likely anaerobic. Moreover, competition for iron uptake may have stressed the cells especially within the balls. Consistent with these observations, fermentation pathway enzymes were up-regulated in *A.act.* in co-culture.

*A.act*. strongly expressed virulence genes both in the presence and absence of *S. mutans*. It was noteworthy that on average 20% of transcriptional activity of this organism was devoted to the *tad* locus producing adhesive fimbriae. The gene encoding leukotoxin was the second most highly expressed gene overall. Thus, in our experimental setup *A. act*. most probably maintained an extremely virulent form. Interestingly, the two genes involved in immune response evasion, encoding catalase KatA and the complement resistance protein ApiA, were repressed in our co-culture, while they were induced in co-culture with *S. gordonii* [28]. This suggests that *A.act.* is more susceptible to the host immune defense in the presence of *S. mutans*, which might explain clinical studies which have found a negative relationship between caries and aggressive periodontitis [50].

Our data show that *S. mutans* probably produces mutacins and is genetically competent in vivo, indicated by the relatively large number of transcripts of *sigX* as well as of many components of the SigX regulon. Moreover, the expression of *comC*, the gene encoding the synthase for the mutacin inducing peptide, was detected in dual species biofilms and in periodontal pockets, but not in biofilms of *S. mutans* alone, neither during exponential growth nor in the stationary phase. Apparently the QS system of *S. mutans* can be induced not only by *C. albicans* and *A. act.*, but also by interaction with diverse members of the oral microbiome. The exact mechanism through which this occurs remains to be elucidated. The data suggest that QS of *S. mutans* is not so much a mechanism for orchestrating gene expression of its own population according to cell density, but a mechanism for adjusting its phenotype to the presence and activity of the oral microbiome. Killing competitors and using their DNA for genetic adaptation provides a powerful survival mechanism in complex communities, but is meaningless in pure culture.

## Conclusion

The complete quorum sensing regulon of *S. mutans* was induced by *A. act.* by an unknown mechanism which required the presence of ComS, the synthase for the XIP prepeptide. *A. act*. grew in a highly virulent form but down-regulated the genes important for escape from the host immune response in co-culture with *S. mutans*. Transformasome and mutacin genes of *S. mutans* were expressed in periodontal pockets, suggesting that its quorum sensing regulon is active in vivo. Because polymicrobial communities harboring streptococci and *Aggregatibacter* spp. are causing oral infectious diseases, the observed interactions may have an important role in the dysbiosis of such communities*.*

## Additional files

Additional file 1: Strains and primers used in the study. (DOCX 26 kb)
Additional file 2: Biofilm formation of *S. mutans* in single and dual-species biofilms with *A. act*. on different media. (a) CDMS, chemically defined medium with sucrose, (b) THBYS, Todd-Hewitt broth with yeast extract and sucrose, (c) YNBB2, biofilm medium (d) AS, artificial saliva, (e) modified AS, (mucin was omitted and haemin was replaced with 1.2 μM FeCl_3_). All media were additionally buffered and supplemented with sucrose. Biofilm mass was determined by crystal violet staining. See methods for details. (TIF 281 kb)
Additional file 3: Activation of the *sigX* promoter of *S. mutans* in single and dual-species biofilms with *A. act* on different media. (a) CDMS, chemically defined medium with sucrose, (b) THBYS, Todd-Hewitt broth with yeast extract and sucrose, (c) YNBB2, biofilm medium (d) AS, artificial saliva, (e) modified AS, (mucin was omitted and haemin was replaced with 1.2 μM FeCl_3_). All media were additionally buffered and supplemented with sucrose. Activation was determined as fluorescence intensity of SMP_*sigX*_GFP, a gfp-reporter for *sigX* expression in *S. mutans.* See methods for details. (TIF 273 kb)
Additional file 4: Influence of mucin on *sigX* fluorescence (A), biofilm formation and *sigX* activation by *A.act.* HK1651 (B,C), and analysis of sequencing depths (D-F). (A) Fluorescence of *S. mutans* reporter strain SMP_*sigX*_GFP co-cultured with *A. act.* in dual-species biofilms on YNBB2 medium supplemented with the indicated amount of mucin. (B) Biofilm biomass and (C) fluorescence of *S. mutans* SMU*sig*XGFP activated by by culture supernatants from single- and dual-species biofilms with *A.act.* HK1651. (D-E) Sequencing depth. Mean log_2_ transformed counts per gene per million reads (CPM) for all detected *S. mutans* (D) and *A. act*. (E) genes. As a cut-off for expression, 10 CPM was chosen. (F) Percentage and number of genes above and below cut-off and with CPM = 0. (TIF 325 kb)
Additional file 5: Sheet 1: Overview of biofilm sequencing results. Sheet 2: Small RNAs expressed by *S. mutans* in dual-species biofilms with *A.act.* Sheet 2: Top 20 genes of *S. mutans* expressed in dual-species biofilms with *A. act*.. Sheet 3: Top 20 genes of *A. act*. expressed in dual-species biofilms with *S. mutans*. Sheet 4: Top 50 most abundant transcripts of *S. mutans* in the periodontal community (sample AU_08, AU_09, AU_12, AU_19). Sheet 5: Relative abundace of transcripts of the SigX regulon of *S. mutans* in the periodontal community (sample AU_8, AU_9, AU_12, AU_19). Sheet 6: Differentially expressed genes of the SigX regulon of *S. mutans* (periodontal pocket versus pure culture at 8 h). Sheet 7: Relative abundance (%) of transcripts of *S. mutans* in four periodontal pocket samples (sample AU_08, AU_09, AU_12, AU_19) Sheet 8: Relative abundance (%) of *A. act.* genes expressed in dual-species biofilms. (XLSX 530 kb)
Additional file 6: C-means clustering of differentially expressed genes of *S. mutans* from dual-species biofilms with *A. act*. during growth in comparison to 4 h. (a) Calculation of minimum centroid distance for a range of cluster numbers. On this basis, 8 clusters were defined (marked with a dashed line). (b) PCA analysis of clusters. Two clear groups are formed. Blue lines indicate relationship between clusters. Changes in expression of genes in group 1 (c), group 2 (d) and cluster 1 to 8 (e) are shown. (TIF 934 kb)
Additional file 7: C-means clustering of differentially expressed genes of *A. act.* from dual-species biofilms with *S. mutans* during growth in comparison to 4 h. (a) Calculation of minimum centroid distance for a range of cluster numbers. On this basis, 8 clusters were defined (marked with a dashed line). (b) PCA analysis of clusters. Two clear groups are formed. Blue lines indicate relationship between clusters. Changes in expression of genes in group 1 (c), group 2 (d) and cluster 1 to 8 (e) are shown. (TIF 807 kb)
Additional file 8: Relative abundance of transcripts from *S. mutans* in metatranscriptomes from periodontal pocket samples. Data are derived from [16–18, 43]. Reads are shown as absolute number of reads in the respective sample (left) and in % of total reads (right). (JPG 1822 kb)
Additional file 9: Relative abundance of transcripts from *A. act.* in metatranscriptomes from periodontal pocket samples. Data are derived from [16–18, 43]. Reads are shown as absolute number of reads in the respective sample (left) and in % of total reads (right). (JPG 1820 kb)
Additional file 10: Microscopic analysis of dual-species biofilms. Dual-species biofilms of *A. act.* and *S. mutans* reporter strain SMP_*sigX*_GFP. (a) *S. mutans* (green) expressed Gfp*, A. act.* (blue) was counterstained with 4′,6-diamidino-2-phenylindole (DAPI). *A. act.* formed “ball-like” clusters of tightly packed cells. (b) Ball-like clusters at higher magnification. (c) Gram staining of biofilms. *S. mutans* (left) appears violet and *A. act.* (right) is stained pink. (d) “Ball-like” clusters of cells at high magnification. Scale bars (a) 20 μm; (b) 100 μm; (c) 50 μm; (e) 6 μm. (a) and (b) were obtained by fluorescence microscopy, (c) by light microscopy, (e) by scanning electron microscopy. (TIF 3732 kb)

## Abbreviations

*A. act*.: *Aggregatibacter actinomycetemcomitans*
AS: Artificial saliva
CDMS: Chemically defined medium with sucrose
ComR: Response regulator induced by XIP
ComS: Autoinducer synthase for the XIP prepeptide
CSP: Competence stimulating peptide
EPS: Extracellular polysaccharide
GFP: Green fluorescent protein
HK: Histidine kinase
MIP (former CSP): Mutacin inducing peptide
PCA: Principal component analysis
q-PCR: Quantitative PCR
q-RT-PCR: Quantitative reverse transcription PCR
QS: quorum sensing
*S.*: *Streptococcus*
SigX: alternative sigma-factor
THBY: Todd-Hewitt broth with yeast extract
THBYS: Todd-Hewitt broth with yeast extract and sucrose
XIP: SigX inducing peptide
XIP: *sigX*-inducing peptide
YNBB2: biofilm medium

## References

[1] Clarke JK (1924). On the bacterial factor in the etiology of dental caries. British journal of experimental pathology.

[2] Loesche WJ (1986). Role of Streptococcus mutans in human dental decay. Microbiol Rev.

[3] Simon-Soro A, Belda-Ferre P, Cabrera-Rubio R, Alcaraz LD, Mira A (2013). A tissue-dependent hypothesis of dental caries. Caries Res.

[4] Simon-Soro A, Guillen-Navarro M, Mira A (2014). Metatranscriptomics reveals overall active bacterial composition in caries lesions. J Oral Microbiol.

[5] Teng F, Yang F, Huang S, Bo C, Xu ZZ, Amir A, Knight R, Ling J, Xu J (2015). Prediction of early childhood caries via spatial-temporal variations of oral microbiota. Cell Host Microbe.

[6] Simon-Soro A, Mira A (2015). Solving the etiology of dental caries. Trends Microbiol.

[7] Guo L, McLean JS, Yang Y, Eckert R, Kaplan CW, Kyme P, Sheikh O, Varnum B, Lux R, Shi W, He X (2015). Precision-guided antimicrobial peptide as a targeted modulator of human microbial ecology. Proc Natl Acad Sci U S A.

[8] Thompson JA, Oliveira RA, Xavier KB (2016). Chemical conversations in the gut microbiota. Gut Microbes.

[9] Guo L, He X, Shi W (2014). Intercellular communications in multispecies oral microbial communities. Front Microbiol.

[10] Cook LC, Federle MJ (2014). Peptide pheromone signaling in Streptococcus and Enterococcus. FEMS Microbiol Rev.

[11] Cook LC, LaSarre B, Federle MJ. Interspecies communication among commensal and pathogenic streptococci. MBio. 2013;4(4):e00382-13. doi:10.1128/mBio.00382-13. 10.1128/mBio.00382-13 PMC3735184 23882015

[12] Aggarwal C, Jimenez JC, Lee H, Chlipala GE, Ratia K, Federle MJ (2015). Identification of quorum-sensing inhibitors disrupting signaling between Rgg and short hydrophobic peptides in streptococci. MBio.

[13] Parashar V, Aggarwal C, Federle MJ, Neiditch MB (2015). Rgg protein structure-function and inhibition by cyclic peptide compounds. Proc Natl Acad Sci U S A.

[14] Chang JC, Jimenez JC, Federle MJ (2015). Induction of a quorum sensing pathway by environmental signals enhances group A streptococcal resistance to lysozyme. Mol Microbiol.

[15] Jorth P, Trivedi U, Rumbaugh K, Whiteley M (2013). Probing bacterial metabolism during infection using high-resolution transcriptomics. J Bacteriol.

[16] Szafranski SP, Deng Z-L, Tomasch J, Jarek M, Bhuju S, Meisinger C, Kühnisch J, Sztajer H, Wagner-Döbler I. Functional biomarkers for chronic periodontitis and insights into the roles of Prevotella nigrescens and Fusobacterium nucleatum: a metatranscriptome analysis. Nature Biofilms and Microbiomes. 2015;1. doi:10.1038/npjbiofilms.2015.17. 10.1038/npjbiofilms.2015.17 PMC5515211 28721234

[17] Duran-Pinedo AE, Chen T, Teles R, Starr JR, Wang X, Krishnan K, Frias-Lopez J (2014). Community-wide transcriptome of the oral microbiome in subjects with and without periodontitis. ISME J.

[18] Yost S, Duran-Pinedo AE, Teles R, Krishnan K, Frias-Lopez J (2015). Functional signatures of oral dysbiosis during periodontitis progression revealed by microbial metatranscriptome analysis. Genome Med.

[19] Mashburn-Warren L, Morrison DA, Federle MJ (2010). A novel double-tryptophan peptide pheromone controls competence in Streptococcus spp. via an Rgg regulator. Mol Microbiol.

[20] Reck M, Tomasch J, Wagner-Dobler I (2015). The alternative sigma factor SigX controls bacteriocin synthesis and competence, the Two quorum sensing regulated traits in streptococcus mutans. PLoS Genet.

[21] Sztajer H, Szafranski SP, Tomasch J, Reck M, Nimtz M, Rohde M, Wagner-Dobler I (2014). Cross-feeding and interkingdom communication in dual-species biofilms of Streptococcus mutans and Candida albicans. ISME J.

[22] Kononen E, Muller HP (2014). Microbiology of aggressive periodontitis. Periodontol.

[23] Hajishengallis G, Lamont RJ (2012). Beyond the red complex and into more complexity: the polymicrobial synergy and dysbiosis (PSD) model of periodontal disease etiology. Mol Oral Microbiol.

[24] Mark Welch JL, Rossetti BJ, Rieken CW, Dewhirst FE, Borisy GG (2016). Biogeography of a human oral microbiome at the micron scale. Proc Natl Acad Sci U S A.

[25] Kolenbrander PE (2011). Multispecies communities: interspecies interactions influence growth on saliva as sole nutritional source. Int J Oral Sci.

[26] Stacy A, McNally L, Darch SE, Brown SP, Whiteley M (2016). The biogeography of polymicrobial infection. Nat Rev Microbiol.

[27] Stacy A, Everett J, Jorth P, Trivedi U, Rumbaugh KP, Whiteley M (2014). Bacterial fight-and-flight responses enhance virulence in a polymicrobial infection. Proc Natl Acad Sci U S A.

[28] Ramsey MM, Whiteley M (2009). Polymicrobial interactions stimulate resistance to host innate immunity through metabolite perception. Proc Natl Acad Sci U S A.

[29] Lemme A, Grobe L, Reck M, Tomasch J, Wagner-Dobler I (2011). Subpopulation-specific transcriptome analysis of competence-stimulating-peptide-induced Streptococcus mutans. J Bacteriol.

[30] Wong L, Sissons C (2001). A comparison of human dental plaque microcosm biofilms grown in an undefined medium and a chemically defined artificial saliva. Arch Oral Biol.

[31] van de Rijn I, Kessler RE (1980). Growth characteristics of group a streptococci in a new chemically defined medium. Infect Immun.

[32] Lau PC, Sung CK, Lee JH, Morrison DA, Cvitkovitch DG (2002). PCR ligation mutagenesis in transformable streptococci: application and efficiency. J Microbiol Methods.

[33] Periasamy S, Kolenbrander PE (2009). Aggregatibacter actinomycetemcomitans builds mutualistic biofilm communities with Fusobacterium nucleatum and Veillonella species in saliva. Infect Immun.

[34] Langmead B, Salzberg SL (2012). Fast gapped-read alignment with Bowtie 2. Nat Methods.

[35] Robinson MD, McCarthy DJ, Smyth GK (2010). edgeR: a Bioconductor package for differential expression analysis of digital gene expression data. Bioinformatics.

[36] Kumar L, Futschik E (2007). Mfuzz: a software package for soft clustering of microarray data. Bioinformation.

[37] McClure R, Balasubramanian D, Sun Y, Bobrovskyy M, Sumby P, Genco CA, Vanderpool CK, Tjaden B (2013). Computational analysis of bacterial RNA-Seq data. Nucleic Acids Res.

[38] Kolde R. pheatmap: Pretty Heatmaps. R package version 0.7.7., version. 2013.

[39] Son M, Ahn SJ, Guo Q, Burne RA, Hagen SJ (2012). Microfluidic study of competence regulation in Streptococcus mutans: environmental inputs modulate bimodal and unimodal expression of comX. Mol Microbiol.

[40] Levesque CM, Mair RW, Perry JA, Lau PC, Li YH, Cvitkovitch DG (2007). Systemic inactivation and phenotypic characterization of two-component systems in expression of Streptococcus mutans virulence properties. Lett Appl Microbiol.

[41] Haubek D, Johansson A. Pathogenicity of the highly leukotoxic JP2 clone of Aggregatibacter actinomycetemcomitans and its geographic dissemination and role in aggressive periodontitis. J Oral Microbiol. 2014;6. doi:10.3402/jom.v6.23980. 10.3402/jom.v6.23980 PMC4139931 25206940

[42] Takashima E, Konishi K (2008). Characterization of a quinol peroxidase mutant in Aggregatibacter actinomycetemcomitans. FEMS Microbiol Lett.

[43] Jorth P, Turner KH, Gumus P, Nizam N, Buduneli N, Whiteley M (2014). Metatranscriptomics of the human oral microbiome during health and disease. MBio.

[44] Szafranski SP, Wos-Oxley ML, Vilchez-Vargas R, Jauregui R, Plumeier I, Klawonn F, Tomasch J, Meisinger C, Kühnisch J, Sztajer H, Pieper DH, Wagner-Döbler I (2015). High-resolution taxonomic profiling of the subgingival microbiome for biomarker discovery and periodontitis diagnosis. Appl Environ Microbiol.

[45] Chong P, Drake L, Biswas I (2008). LiaS regulates virulence factor expression in Streptococcus mutans. Infect Immun.

[46] Shankar M, Mohapatra SS, Biswas S, Biswas I (2015). Gene regulation by the LiaSR Two-component system in streptococcus mutans. PLoS One.

[47] HW v’t, Veerman EC, Nieuw Amerongen AV, Ligtenberg AJ (2014). Antimicrobial defense systems in saliva. Monogr Oral Sci.

[48] van der Hoeven JS, van den Kieboom CW, Camp PJ (1990). Utilization of mucin by oral Streptococcus species. Antonie Van Leeuwenhoek.

[49] Wessel AK, Arshad TA, Fitzpatrick M, Connell JL, Bonnecaze RT, Shear JB, Whiteley M (2014). Oxygen limitation within a bacterial aggregate. MBio.

[50] Sulugodu RS (2014). Low levels of caries in aggressive periodontitis: a literature review. Saudi Dent J.

