# Supplementary material for: Quorum sensing of Streptococcus mutans is activated by Aggregatibacter actinomycetemcomitans and by the periodontal microbiome
Source: BMC Genomics. 2017 Mar 20;18:238. doi: 10.1186/s12864-017-3618-5 (PMC5359896; doi:10.1186/s12864-017-3618-5)
Supplement: Additional file 1: — Strains and primers used in the study. (DOCX 26 kb) [file 12864_2017_3618_MOESM1_ESM.docx]

**Table S1 Strains and primers used in this study**

| **Strain name** | **Characterisitcs** | **Source/Reference** |
| --- | --- | --- |
| *Aggregatibacter actinomycetemcomitans* ATCC33384 | smooth, low leukotoxic | G. Conrads, RWTH Aachen |
| *Aggregatibacter actinomycetemcomitans* HK1651 | rough, high leukotoxic | CCUG56173 |
| *Streptococcus mutans UA159* | wild-type | ATCC700610 |
| *S. mutans SMPsigXGFP* | UA159 ϕ(sigX_p_-gfp), Erm r | Lemme et al., 2011 |
| *S. mutans ΔbrsRM* | UA159 ΔSMU.2080-2081::Erm r | This study |
| *S. mutans ΔciaH* | UA159 ΔSMU.1128::Erm r | Levesque et al., 2007 |
| *S. mutans ΔcomD* | UA159 ΔSMU.1916::Erm r | Levesque et al., 2007 |
| *S. mutans ΔcomE* | UA159 ΔSMU.1917::Erm r | Reck et al., 2011 |
| *S. mutans ΔcomS* | UA159 ΔNC_004350.2 (62613 - 62666)::Erm r | Sztajer et al., 2014 |
| *S. mutans ΔcovS* | UA159 ΔSMU.1145::Erm r | Levesque et al., 2007 |
| *S. mutans ΔhdrRM* | UA159 ΔSMU.1854-1855::Erm r | This study |
| *S. mutans ΔHK12* | UA159 ΔSMU.1548::Erm r | Levesque et al., 2007 |
| *S. mutans ΔkinF* | UA159 ΔSMU.928::Erm r | Levesque et al., 2007 |
| *S. mutans ΔkinG* | UA159 ΔSMU.1009::Erm r | Levesque et al., 2007 |
| *S. mutans ΔlevS* | UA159 ΔSMU.1965::Erm r | Levesque et al., 2007 |
| *S. mutans ΔliaS* | UA159 ΔSMU.468::Erm r | Levesque et al., 2007 |
| *S. mutans ΔliaS'* | UA159 ΔSMU.468::Erm r | This study |
| *S. mutans ΔlytS* | UA159 ΔSMU.577::Erm r | Levesque et al., 2007 |
| *S. mutans ΔnlmAB* | UA159 ΔSMU.150-151::Erm r | M. Reck, HZI Braunschweig |
| *S. mutans ΔnlmC* | UA159 ΔSMU.1914c::Erm r | Reck et al., 2011 |
| *S. mutans ΔphoR* | UA159 ΔSMU.1037::Erm r | Levesque et al., 2007 |
| *S. mutans ΔscnK* | UA159 ΔSMU.1814::Erm r | Levesque et al., 2007 |
| *S. mutans ΔsigX* | UA159 ΔSMU.1997::Erm r | Reck et al., 2011 |
| *S. mutans ΔspaK* | UA159 ΔSMU.660::Erm r | Levesque et al., 2007 |
| *S. mutans ΔvicK* | UA159 ΔSMU.1516::Erm r | Levesque et al., 2007 |
| **Primer** | **Sequence** |  |
| BrsRMP1 | TAACATCAGCCTGCTCAACTAGCAT |  |
| BrsRMP2 | AGGCGCGCCCAGTTATCATCCTTTCTTGATTTTAGTATATAG |  |
| BrsRMP3 | ATTGGCCGGCCAGGCAGATGCAAGTTTGATGG |  |
| BrsRMP4 | TTGGCTCCCTTTTGGCTGTATTTTAAAC |  |
| ERMFor | ACCGGGCCCAAAATTTGTTTGAT |  |
| ERMRev | ATTGGCCGGCCAGTCGGCAGCGACTCATAGAAT |  |
| HdrRMP1 | CACAAACAGGAGAAAACACTCATGGATG |  |
| HdrRMP2 | AGGCGCGCCATGTCTAAGAGGTAACCATTAGTGATAAATG |  |
| HdrRMP3 | ATTGGCCGGCCGATATCTTACGCCTTTATTAGTAAATAATGCATG |  |
| HdrRMP4 | AAGGAGGGCAGACTAATGAACAGG |  |
| LiaSP1 | GAACTGAAGGAAGCCTTATGCG |  |
| LiaSP2 | AGGCGCGCCGGAAATGATGGTAACTGCCG |  |
| LiaSP3 | ATTGGCCGGCCAGTGCTGAAAATAAGGGGGTTG |  |
| LiaSP4 | GCATAGACAACGGCTTGGGTC |  |

rences: (1-4)

**Reference List**

1. **Lemme, A., L. Grobe, M. Reck, J. Tomasch, and I. Wagner-Dobler**. 2011. Subpopulation-specific transcriptome analysis of competence-stimulating-peptide-induced Streptococcus mutans. J.Bacteriol. **193**:1863-1877. doi:JB.01363-10 [pii];10.1128/JB.01363-10 [doi].

2. **Levesque, C. M., R. W. Mair, J. A. Perry, P. C. Lau, Y. H. Li, and D. G. Cvitkovitch**. 2007. Systemic inactivation and phenotypic characterization of two-component systems in expression of Streptococcus mutans virulence properties. Lett.Appl.Microbiol. **45**:398-404. doi:LAM2203 [pii];10.1111/j.1472-765X.2007.02203.x [doi].

3. **Reck, M., K. Rutz, B. Kunze, J. Tomasch, S. K. Surapaneni, S. Schulz, and I. Wagner-Dobler**. 2011. The biofilm inhibitor carolacton disturbs membrane integrity and cell division of Streptococcus mutans through the serine/threonine protein kinase PknB. J.Bacteriol. **193**:5692-5706. doi:JB.05424-11 [pii];10.1128/JB.05424-11 [doi].

4. **Sztajer, H., S. P. Szafranski, J. Tomasch, M. Reck, M. Nimtz, M. Rohde, and I. Wagner-Dobler**. 2014. Cross-feeding and interkingdom communication in dual-species biofilms of Streptococcus mutans and Candida albicans. ISME.J. **8**:2256-2271. doi:ismej201473 [pii];10.1038/ismej.2014.73 [doi].
