# Supplementary figures and images for: Quorum sensing of Streptococcus mutans is activated by Aggregatibacter actinomycetemcomitans and by the periodontal microbiome
Source: BMC Genomics. 2017 Mar 20;18:238. doi: 10.1186/s12864-017-3618-5 (PMC5359896; doi:10.1186/s12864-017-3618-5)

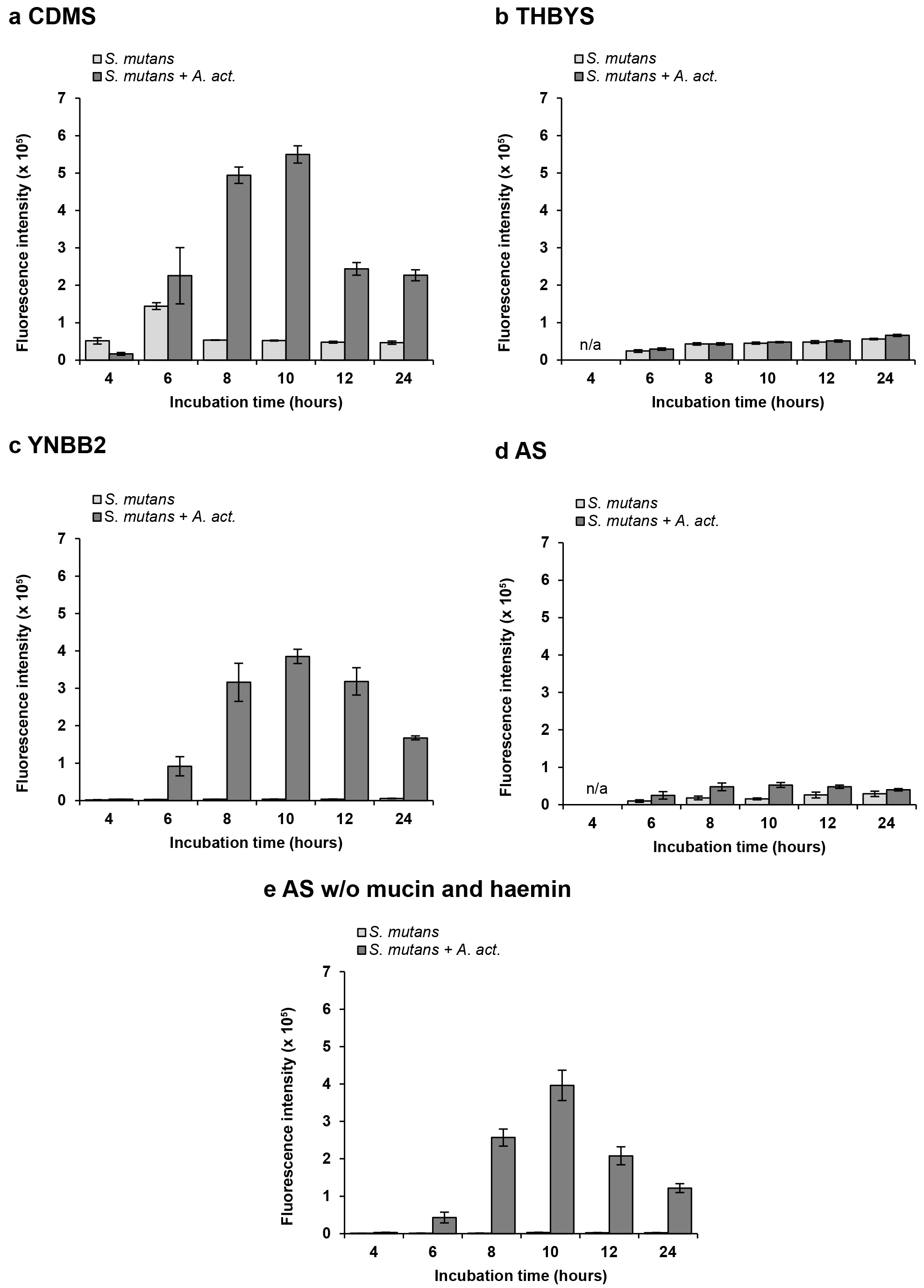

Supplement: Additional file 2: — Biofilm formation of S. mutans in single and dual-species biofilms with A. act. on different media. (a) CDMS, chemically defined medium with sucrose, (b) THBYS, Todd-Hewitt broth with yeast extract and sucrose, (c) YNBB2, biofilm medium (d) AS, artificial saliva, (e) modified AS, (mucin was omitted and haemin was replaced with 1.2 μM FeCl3). All media were additionally buffered and supplemented with sucrose. Biofilm mass was determined by crystal violet staining. See methods for details. (TIF 281 kb) [file 12864_2017_3618_MOESM2_ESM.tif]

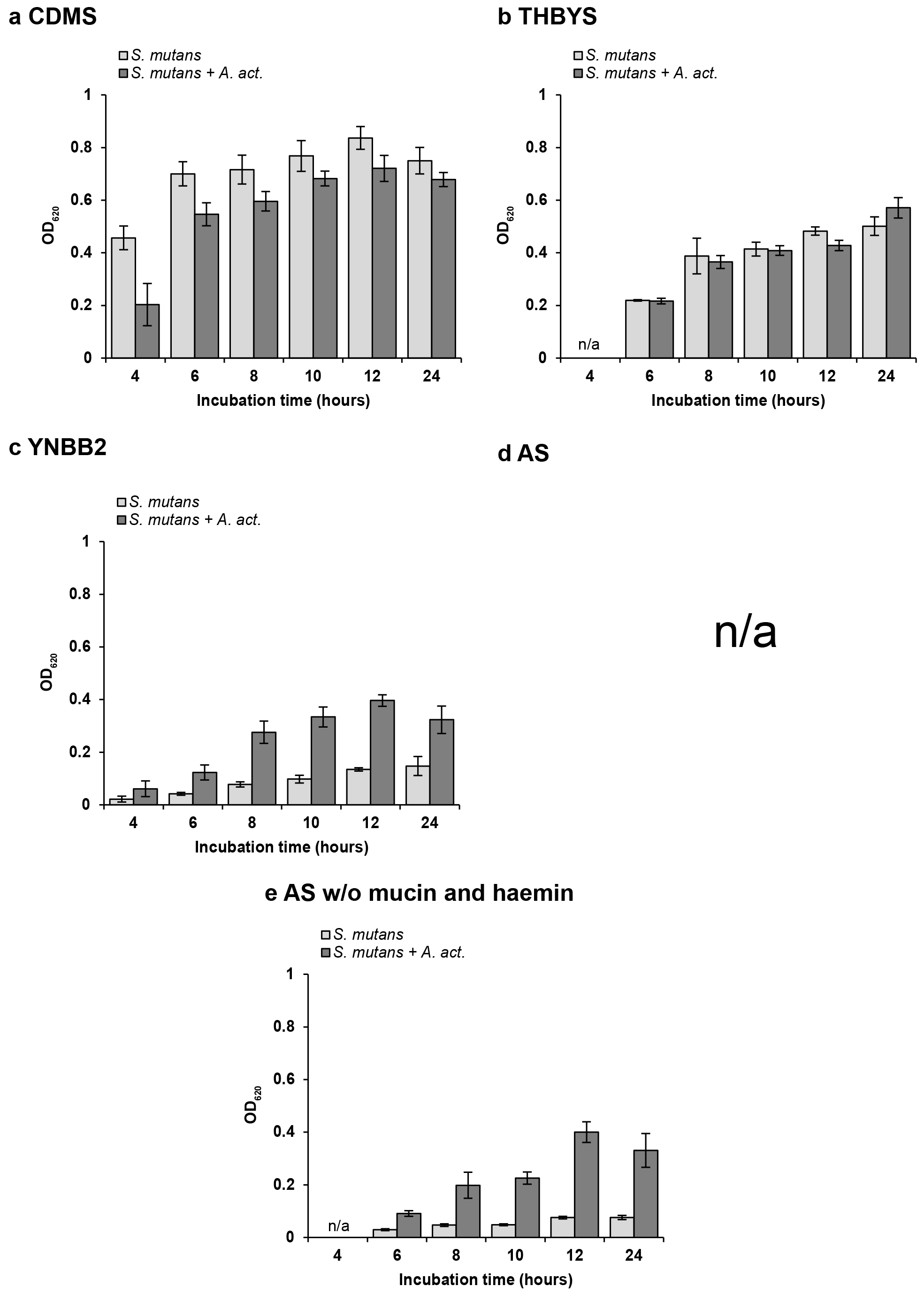

Supplement: Additional file 3: — Activation of the sigX promoter of S. mutans in single and dual-species biofilms with A. act on different media. (a) CDMS, chemically defined medium with sucrose, (b) THBYS, Todd-Hewitt broth with yeast extract and sucrose, (c) YNBB2, biofilm medium (d) AS, artificial saliva, (e) modified AS, (mucin was omitted and haemin was replaced with 1.2 μM FeCl3). All media were additionally buffered and supplemented with sucrose. Activation was determined as fluorescence intensity of SMPsigXGFP, a gfp-reporter for sigX expression in S. mutans. See methods for details. (TIF 273 kb) [file 12864_2017_3618_MOESM3_ESM.tif]

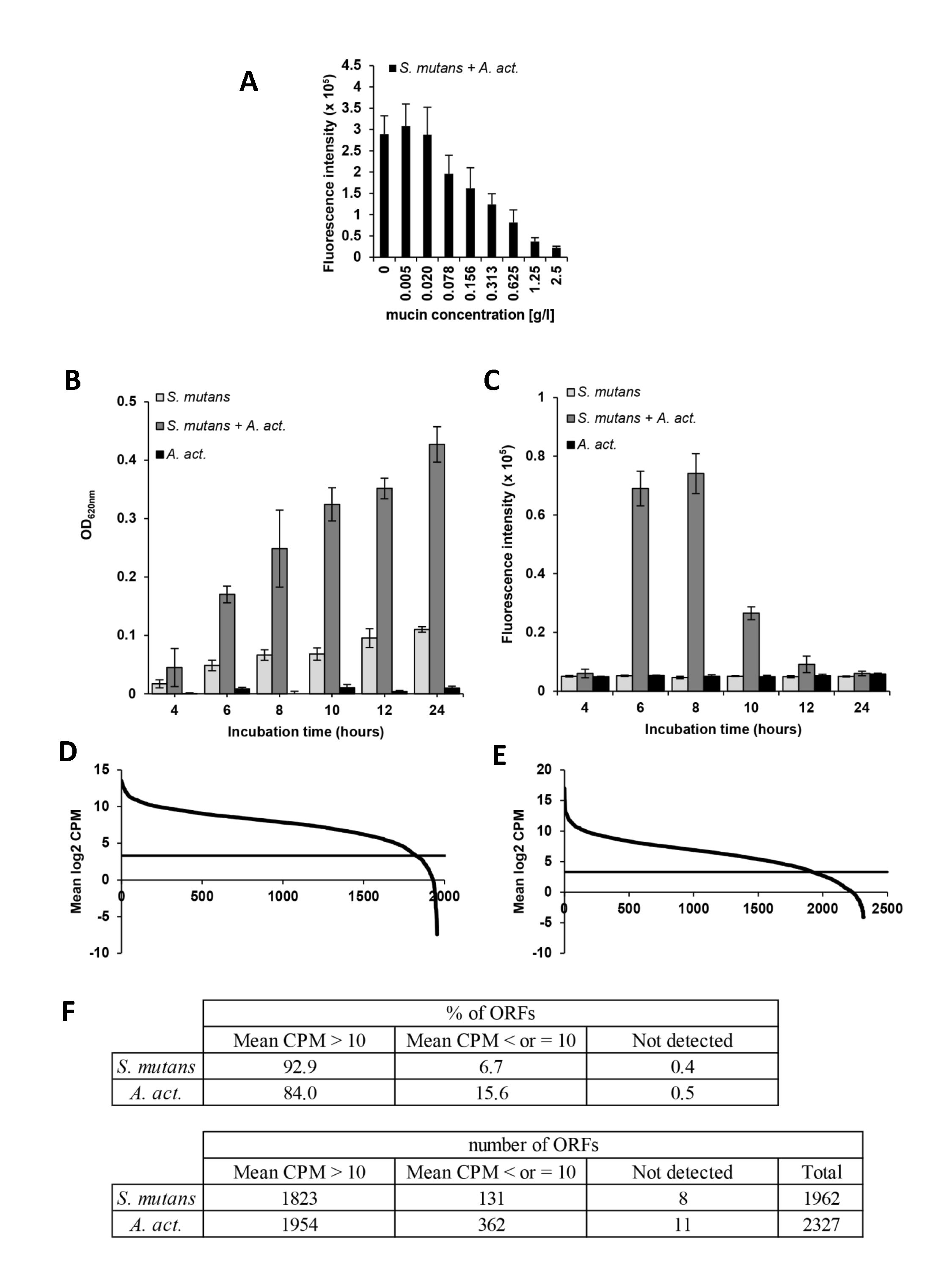

Supplement: Additional file 4: — Influence of mucin on sigX fluorescence (A), biofilm formation and sigX activation by A.act. HK1651 (B,C), and analysis of sequencing depths (D-F). (A) Fluorescence of S. mutans reporter strain SMPsigXGFP co-cultured with A. act. in dual-species biofilms on YNBB2 medium supplemented with the indicated amount of mucin. (B) Biofilm biomass and (C) fluorescence of S. mutans SMUsigXGFP activated by by culture supernatants from single- and dual-species biofilms with A.act. HK1651. (D-E) Sequencing depth. Mean log2 transformed counts per gene per million reads (CPM) for all detected S. mutans (D) and A. act. (E) genes. As a cut-off for expression, 10 CPM was chosen. (F) Percentage and number of genes above and below cut-off and with CPM = 0. (TIF 325 kb) [file 12864_2017_3618_MOESM4_ESM.tif]

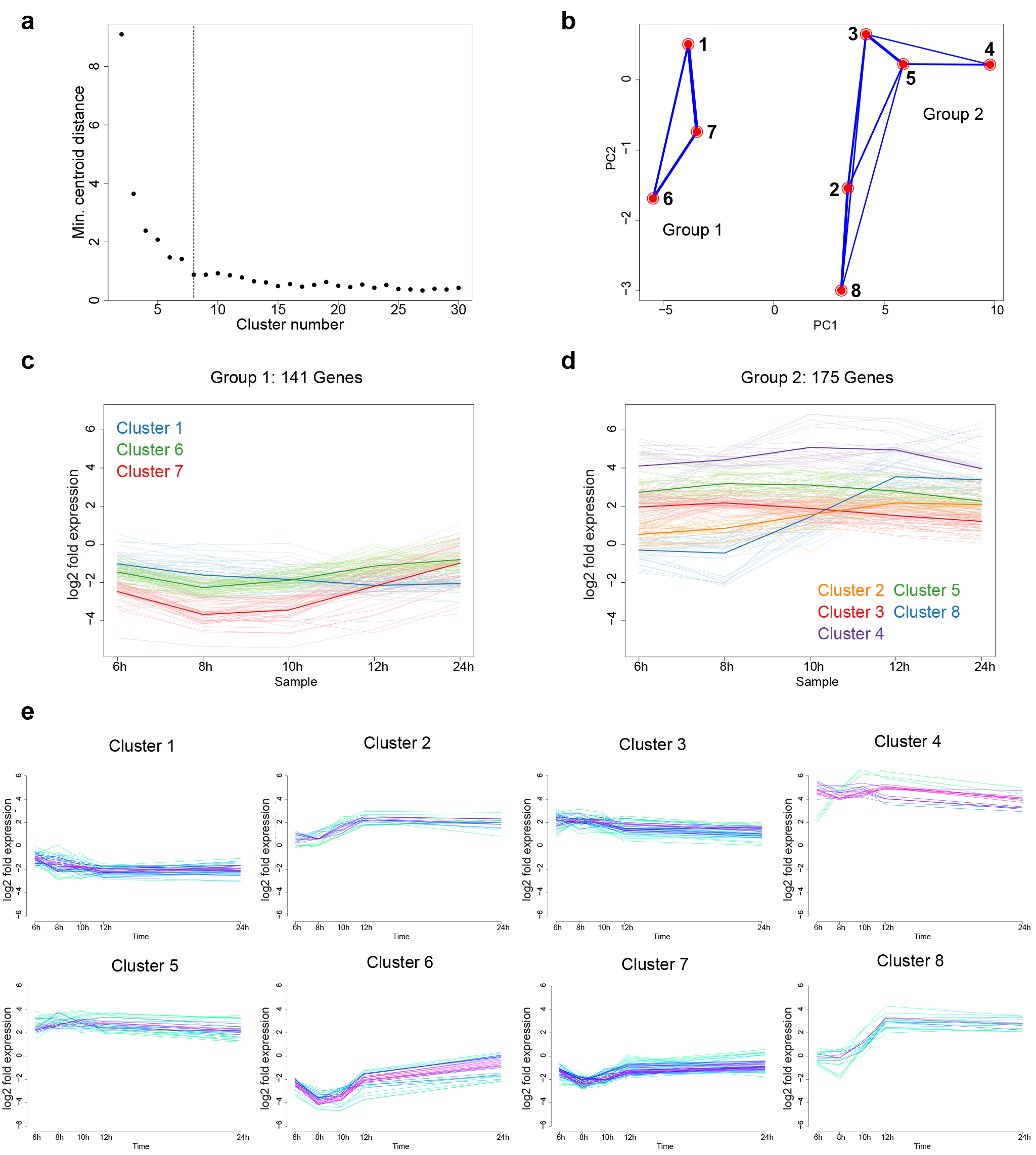

Supplement: Additional file 6: — C-means clustering of differentially expressed genes of S. mutans from dual-species biofilms with A. act. during growth in comparison to 4 h. (a) Calculation of minimum centroid distance for a range of cluster numbers. On this basis, 8 clusters were defined (marked with a dashed line). (b) PCA analysis of clusters. Two clear groups are formed. Blue lines indicate relationship between clusters. Changes in expression of genes in group 1 (c), group 2 (d) and cluster 1 to 8 (e) are shown. (TIF 934 kb) [file 12864_2017_3618_MOESM6_ESM.tif]

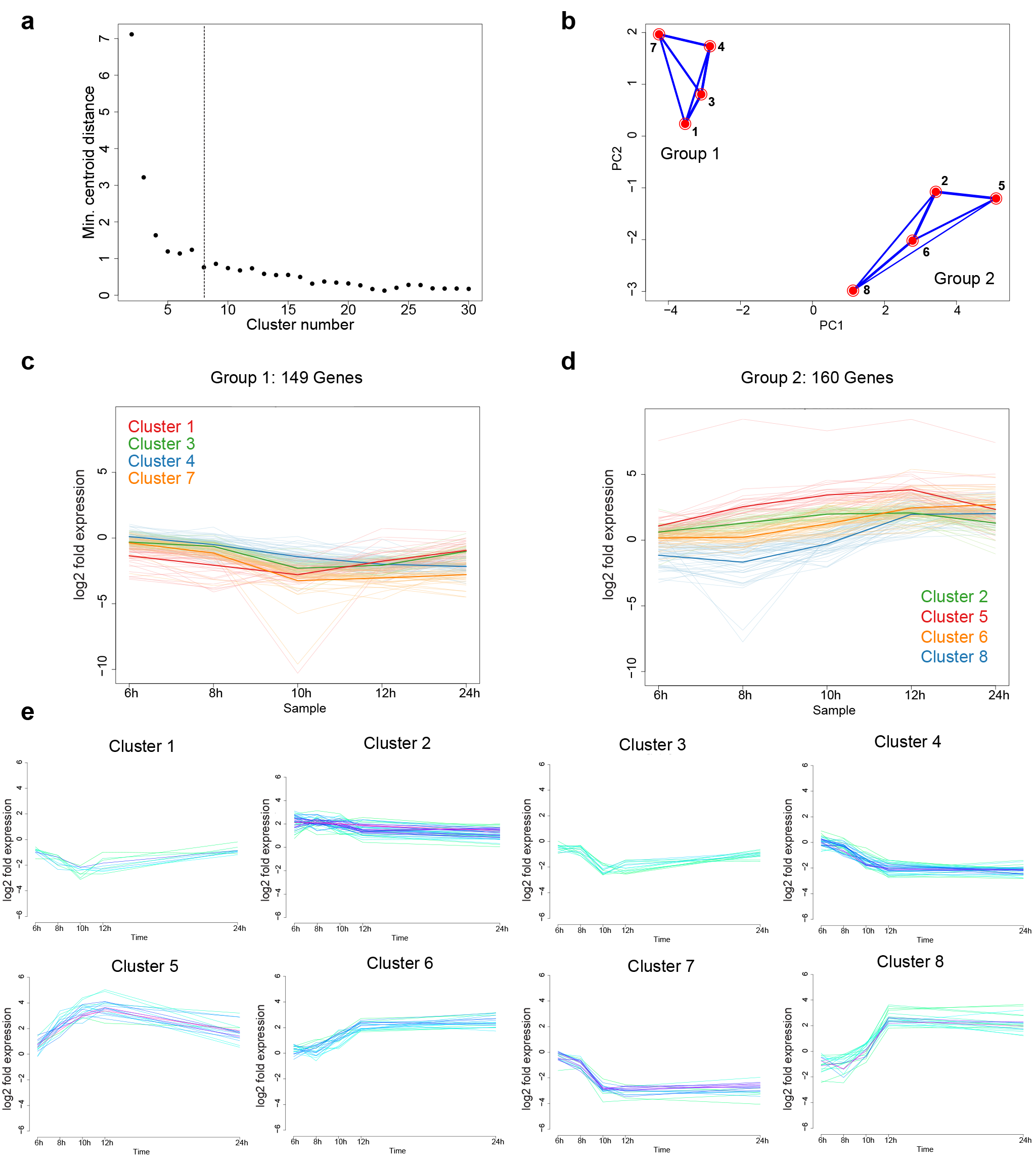

Supplement: Additional file 7: — C-means clustering of differentially expressed genes of A. act. from dual-species biofilms with S. mutans during growth in comparison to 4 h. (a) Calculation of minimum centroid distance for a range of cluster numbers. On this basis, 8 clusters were defined (marked with a dashed line). (b) PCA analysis of clusters. Two clear groups are formed. Blue lines indicate relationship between clusters. Changes in expression of genes in group 1 (c), group 2 (d) and cluster 1 to 8 (e) are shown. (TIF 807 kb) [file 12864_2017_3618_MOESM7_ESM.tif]

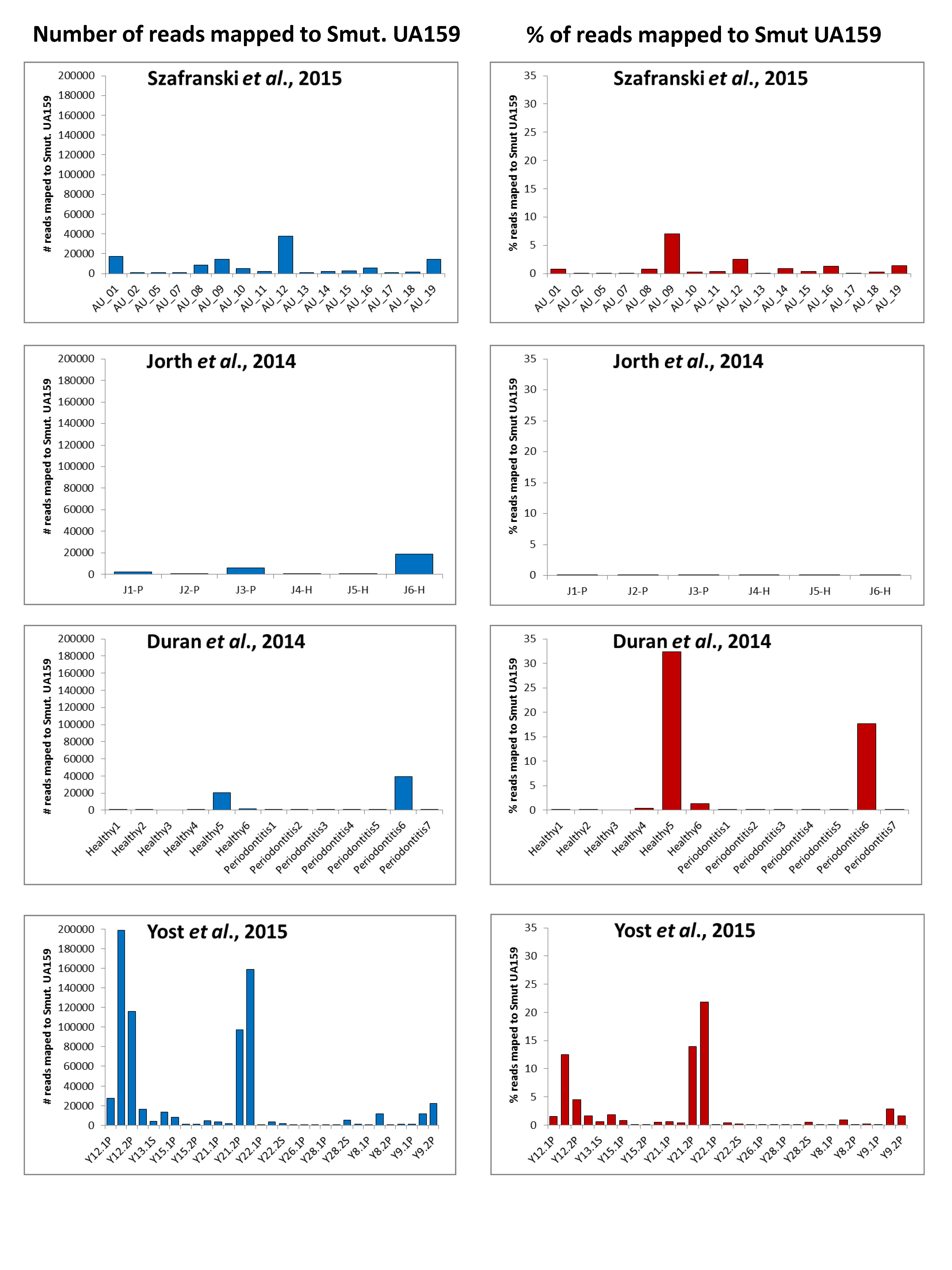

Supplement: Additional file 8: — Relative abundance of transcripts from S. mutans in metatranscriptomes from periodontal pocket samples. Data are derived from [16–18, 43]. Reads are shown as absolute number of reads in the respective sample (left) and in % of total reads (right). (JPG 1822 kb) [file 12864_2017_3618_MOESM8_ESM.jpg]

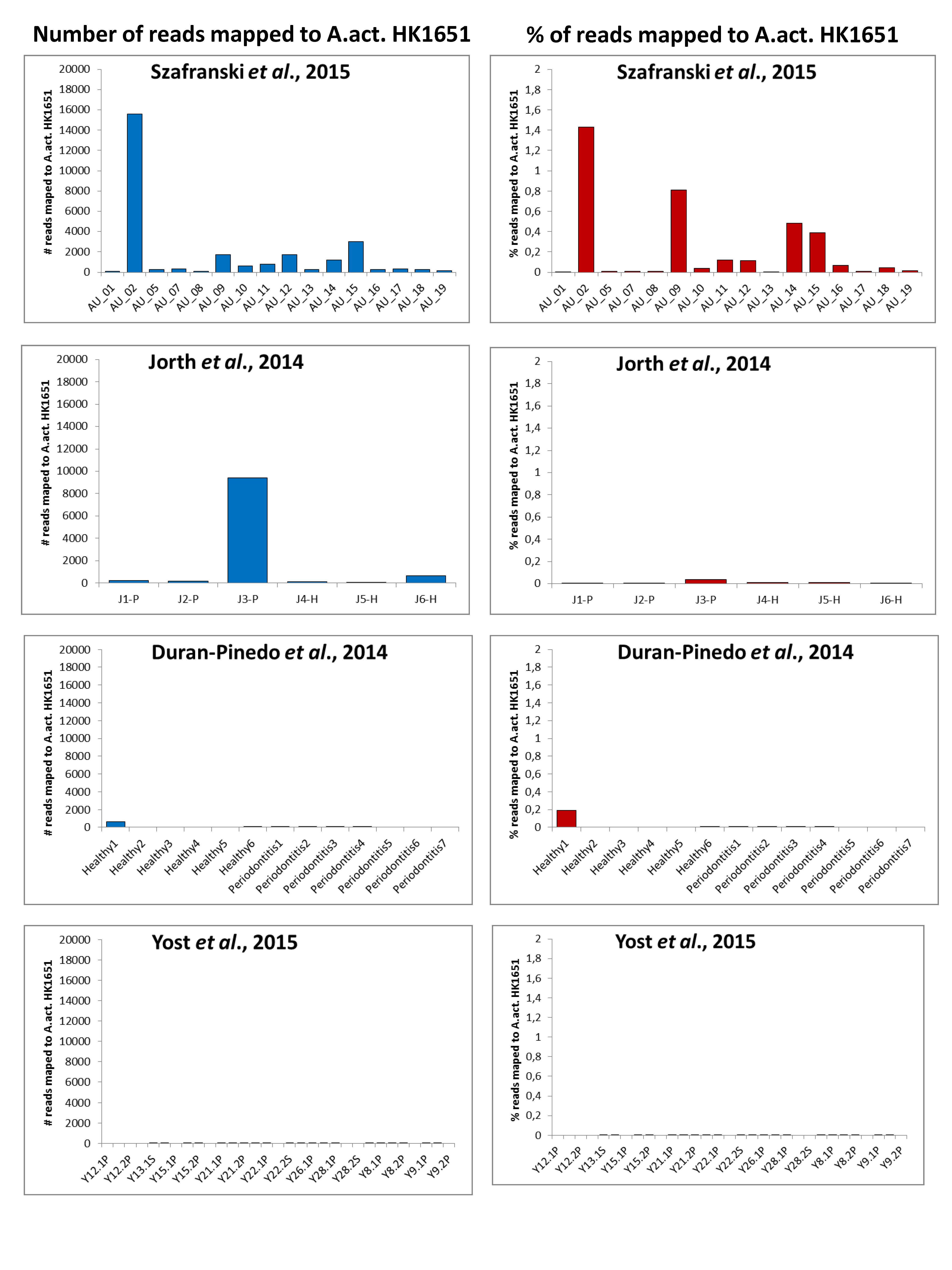

Supplement: Additional file 9: — Relative abundance of transcripts from A. act. in metatranscriptomes from periodontal pocket samples. Data are derived from [16–18, 43]. Reads are shown as absolute number of reads in the respective sample (left) and in % of total reads (right). (JPG 1820 kb) [file 12864_2017_3618_MOESM9_ESM.jpg]

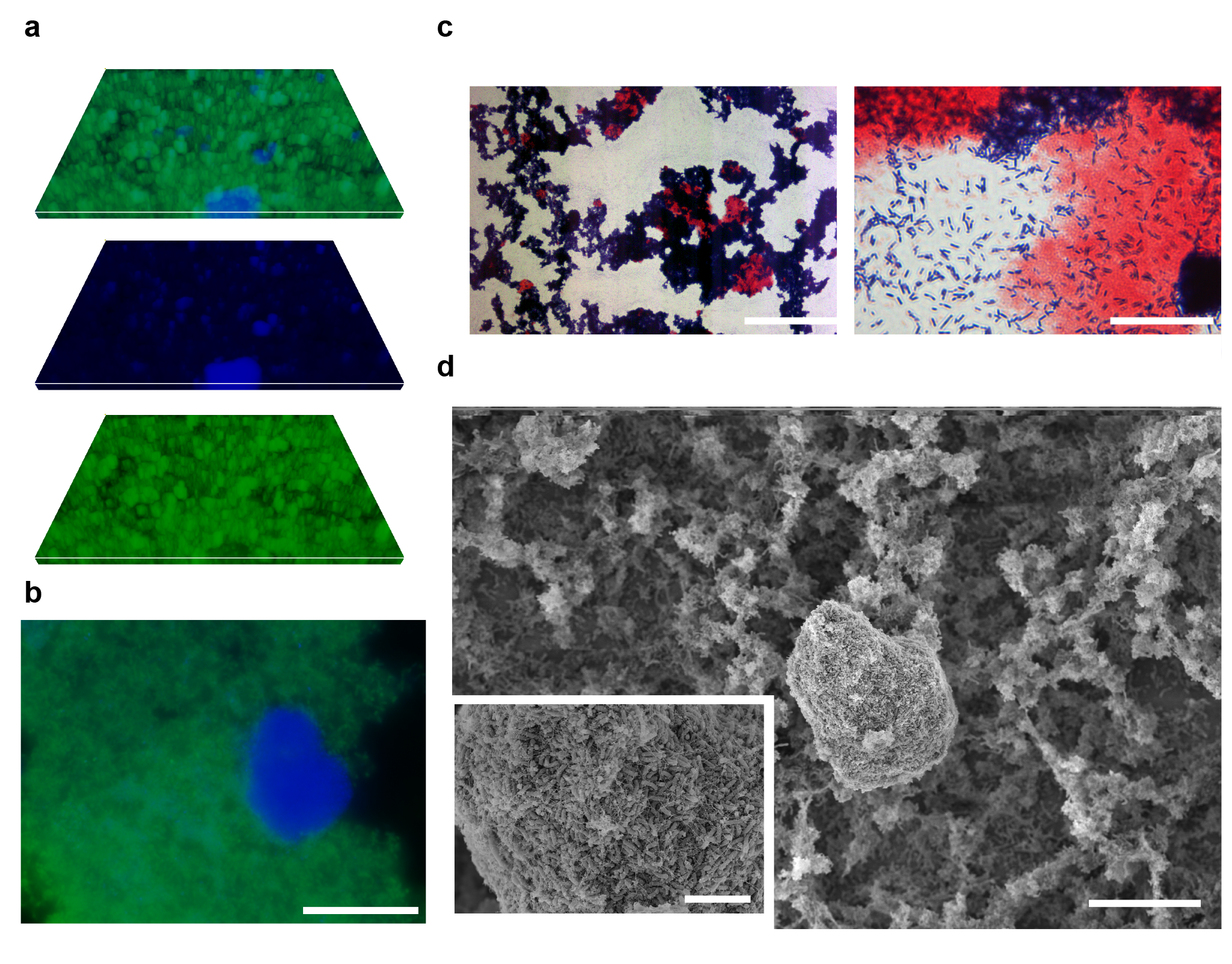

Supplement: Additional file 10: — Microscopic analysis of dual-species biofilms. Dual-species biofilms of A. act. and S. mutans reporter strain SMPsigXGFP. (a) S. mutans (green) expressed Gfp, A. act. (blue) was counterstained with 4′,6-diamidino-2-phenylindole (DAPI). A. act. formed “ball-like” clusters of tightly packed cells. (b) Ball-like clusters at higher magnification. (c) Gram staining of biofilms. S. mutans (left) appears violet and A. act. (right) is stained pink. (d) “Ball-like” clusters of cells at high magnification. Scale bars (a) 20 μm; (b) 100 μm; (c) 50 μm; (e) 6 μm. (a) and (b) were obtained by fluorescence microscopy, (c) by light microscopy, (e) by scanning electron microscopy. (TIF 3732 kb) [file 12864_2017_3618_MOESM10_ESM.tif]
